# Analysis of complement system and its related factors in Alzheimer’s disease

**DOI:** 10.1186/s12883-023-03503-0

**Published:** 2023-12-19

**Authors:** Xi-Chen Zhu, Bin-Feng Tang, Meng-Zhuo Zhu, Jing Lu, Han-Xiao Lin, Jia-Ming Tang, Rong Li, Tao Ma

**Affiliations:** 1 Department of Neurology, The Wuxi No. 2 People’s Hospital, Jiangnan University Medical Center, Wuxi, Jiangsu Province China; 2 Brain Institue, Jiangnan University, Wuxi, Jiangsu Province China; 3 Department of Neurology, The Wuxi No. 2 People’s Hospital, Affiliated Wuxi Clinical College of Nantong University, Wuxi, Jiangsu Province China; 4 Department of Neurology, The Affiliated Wuxi No. 2 People’s Hospital of Nanjing Medical University, No. 68 Zhongshan Road, Wuxi, Jiangsu 214000 China; 5 Department of Pharmacy, The Affiliated Wuxi No. 2 People’s Hospital, Jiangnan University Medical Center, Wuxi, Jiangsu Province China

**Keywords:** Alzheimer’s disease, Complement system, Gene, Protein, Signaling pathway, Bioinformatics analysis

## Abstract

Alzheimer’s disease (AD) is a primary cause of dementia. The complement system is closely related to AD pathology and may be a potential target for the prevention and treatment of AD. In our study, we conducted a bioinformatics analysis to analyze the role of the complement system and its related factors in AD using Gene Expression Omnibus (GEO) data. We also conducted a functional analysis. Our study verified that 23 genes were closely related to differentially expressed complement system genes in diseases after intersecting the disease-related complement system module genes and differentially expressed genes. The STRING database was used to predict the interactions between the modular gene proteins of the differential complement system. A total of 21 gene proteins and 44 interaction pairs showed close interactions. We screened key genes and created a diagnostic model. The predictive effect of the model was constructed using GSE5281 and our study indicated that the predictive effect of the model was good. Our study also showed enriched negative regulation of Notch signaling, cytokine secretion involved in the immune response pathway, and cytokine secretion involved in immune response hormone-mediated apoptotic signaling pathway. We hope that our study provides a promising target to prevent and delay the onset, diagnosis, and treatment of AD.

**Supplementary Information:**

The online version contains supplementary material available at 10.1186/s12883-023-03503-0.

## Introduction

Alzheimer’s disease (AD) is a primary cause of dementia. The number of patients with AD is expected to reach 100 million by 2050. AD has become one of the most expensive, deadliest, and burdensome diseases of this century [1]. Therefore, it is important to understand the pathogenesis of AD and identify effective treatment methods.

At present, there are many hypotheses about the pathogenesis of AD such as: β-amyloid protein (Aβ) hypothesis, tau protein hypothesis, central cholinergic injury hypothesis, excitatory amino acid toxicity hypothesis, and neuroimmunity hypothesis. Neural immunity has been proven to be closely related to the pathology of AD, and its important components: immune cells (such as T cells [2] and B cells [3]), and various immune active substances (such as complement system-related molecules [4] and neuroinflammatory factors [5]), have been reported to be involved in the pathogenesis of AD. The complement system is an important part of the neuroimmune system and participates in multiple pathological processes in AD. C3 [6], complement receptor 1 (CR1) [7], C1q [8], and C3aR [6] are involved in Aβ metabolism. C4 [9], C3 [10, 11], and CR1 [12] were involved in tau pathology. C1q [13] and CR1 [12] are involved in neuroinflammation. C1q [8], C3 [8], and CR1 [12] are involved in synaptic formation. In conclusion, the complement system is closely related to AD pathology and may be a potential target for the prevention and treatment of AD.

As we all know, many factors are also involved in the complement system to regulate the pathological process of AD. Examining literatures from these years, we have found that the complement system can modulate nucleotide binding oligomerization domain leucine rich repeat and pyrin domain containing 3 inflammasome [14], triggering receptor expressed on myeloid cells 2 [15], and several inflammation-related factors such as tumor necrosis factor [16, 17], interleukin-1 [16, 17], interleukin-6 [16, 17], C-reactive protein [17], and interleukin-10 [16]. The above research suggests that the complement system may be involved in the pathology of AD through its associated genes, proteins, and related signaling pathway. However, there is currently no literature reporting on this aspect. Hence, in the present study, we focused on identifying the role of the complement system and its key related genes, proteins and signaling pathways in AD. We conducted a bioinformatics analysis to analyze the role of the complement system and its related factors in AD. Moreover, functional enrichment analyses and a diagnostic prediction model were also carried out in our study. We hope that our study provides a promising target to prevent and delay the onset, diagnosis, and treatment of AD.

## Methods

### Data acquisition

The datasets generated and analyzed in the current study are available in the NCBI GEO repository (http://www.ncbi.nlm.nih.gov/geo/) under the accession numbers (GSE109887 and GSE5281). The dataset originated from the middle temporal gyrus tissue. Our primary criterion for sample selection was the dataset sample size, followed by gene quantity in the gene expression matrix of the dataset. Through GEO database retrieval, we identified that GSE109887 had the largest sample size from middle temporal gyrus tissue and a more abundant gene quantity in its expression matrix. Consequently, we designated it as the primary dataset for analysis (training set); with GSE5281, featuring fewer samples and genes, serving as the validation set. Both GSE109887 and GSE5281 are datasets used for Expression Profiling by Array [18]. Data on AD patients data are available from the NCBI GEO database [19] (http://www.ncbi.nlm.nih.gov/geo/), and the serial number of the AD patients’ dataset is GSE109887. The data included samples of middle temporal gyrus tissue from 46 patients with AD and 32 healthy controls without AD. The gene microarray was determined using the GPL10904 Illumina HumanHT-12 V4.0 expression bead-chip (gene symbol) platform. This dataset was primarily treated as an analytical dataset.

In addition, we downloaded the AD patients’ validation dataset from the NCBI GEO database. The serial number of the AD patient data was GSE5281. The data included samples of middle temporal gyrus tissue from 16 patients with AD and 12 healthy controls without AD. Gene microarray analysis was performed using the GPL570 (HG-U133_Plus_2) Affymetrix Human Genome U133 Plus 2.0 Array. This dataset was primarily treated as an analytical dataset to validate the diagnostic models.

When conducting data analysis, we performed an analysis of abnormal/outlier samples on the training set data. As shown in the figure below, all samples, as represented by box plots and PCA analysis charts, indicate that the analyzed data set does not contain any abnormal samples (Supplementary Fig. 1).

### Data preprocessing

For the above two datasets, firstly, we downloaded preprocessed, standardized, and log2-transformed probe expression matrices from the GEO database. Secondly, we obtained annotation files for the platforms and conducted a one-to-one matching between probe numbers and Gene symbols. Probes that did not have a matching Gene symbol were excluded. In cases where different probes mapped to the same gene, we calculated the mean expression value of these different probes to represent the final expression value for that gene, which was utilized in subsequent analyses. After preprocessing, GSE109887 yielded an expression matrix of 31,700 genes across 78 samples; whereas GSE5281 produced an expression matrix of 23,520 genes across 28 samples.

### Differential gene analysis

Based on the analysis dataset, we employed the limma package [20] (Version 3.10.3, http://www.bioconductor.org/packages/2.9/bioc/html/limma.html) and its classical Bayesian methods to analyze differential gene expression between AD and Control groups. All genes underwent analysis, resulting in corresponding *P* values and logFC values. Additionally, the BH correction was applied to obtain adjusted *P*-values (adj.p.val). We evaluated at two levels: differential fold change and significance. The threshold for differential expression was set as adj.p.val < 0.05 and |logFC|> 0.585. After identifying differentially expressed genes, a volcano plot was generated, and expression distribution heatmaps between normal and disease groups were separately illustrated [21–23]. After identifying differentially expressed genes, a volcano plot was generated, and expression distribution heatmaps between normal and disease groups were separately illustrated.

### WGCNA (Weighted Gene Co-expression Network analysis) was used to analyze disease-associated complement system related gene modules

We conducted a search in the CTD database [24] (https://ctdbase.org/) using the keyword “complement system” to retrieve genes related to the complement system. In addition, Additionally, a similar search was performed in GeneCards [25] (https://www.genecards.org/) using the keyword “complement system”, with a threshold set at Relevance score >= 10 [26, 27], to obtain complement system-related genes. The genes obtained from both databases were merged, removing duplicates, and then matched with the genes in the analysis dataset. The resulting expression matrix of complement system genes was utilized for WGCNA.

We utilized the R package WGCNA [28] (Version 1.61, https://cran.r-project.org/web/packages/WGCNA/) to analyze the complement system genes, aiming to identify modules of genes that exhibit high levels of co-expression variation.

In the WGCNA algorithm, the elements of the gene co-expression matrix are weighted values representing the correlation coefficients between genes. The criterion for selecting these weights is to ensure that the connections between genes within each gene network follow a scale-free network distribution. The specific weighted values are referred to as the soft power. Initially, we set a series of powers and then calculated the square value of the correlation coefficient between connectivity k, p (k), and the average connectivity under each power value. An appropriate power value is then selected to achieve a scale-free network distribution among genes in the network. Subsequently, parameters were set based on clustering and dynamic pruning (minModuleSize = 30: each module contained at least 30 genes; MEDissThres = 0.25: combining modules whose similarity degree was greater than 0.75). Genes with a high correlation were clustered into modules, and the correlation between modules and phenotypes was calculated. Here, the phenotype refers to whether the samples were diseased or normal. Modules with absolute correlation coefficients greater than 0.3 and significance values less than 0.05 were selected as modules closely related to diseases.

### Acquisition of disease-related differential complement system genes

Taking the intersection of the disease-related complement system module genes (method 2.4) and differential genes (method 2.3) obtained above, further identify differential complement system genes closely associated with the disease.

### Interaction network analysis and co-expression relationship analysis of corresponding proteins of genes in the differential complement system

In order to understand the protein-protein interaction (PPI) relationships among the differentially expressed complement system genes obtained above, we utilized the online STRING database [29] (Version: 11.0, http://www.string-db.org/) for predicting and analyzing potential interactions between the proteins encoded by these genes. The species was set to Homo sapiens (homo), and the PPI score threshold was set to 0.4 (medium confidence).

In addition, Pearson’s correlation coefficients were calculated between gene pairs in the differential complement system to observe the relationships between gene co-expression trends.

### Key gene screening and diagnostic model construction

Based on the differential complement system genes obtained above, we first calculated the diagnostic AUC value of each gene using the expression value of each gene in each sample, combined with the grouping information of the sample, and selected the gene with an AUC > 0.8 as the candidate gene related to the disease. Subsequently, the LASSO (least absolute shrinkage and selection operator) algorithm was used to screen the feature genes. Glmnet package Version 4.0-2 using R language 3.6.1 [20] (https://cran.r-project.org/web/packages/glmnet/index.html) for a preliminary screening of candidate genes associated with disease regression analysis The parameter was set to nfold = 20, that is, 20-fold cross-validation was carried out to screen characteristic genes, and the following formula was used to construct the model:

$$\mathrm{Riskscore }= \sum {\upbeta }_{{\text{gene}}}\times {{\text{Exp}}}_{{\text{gene}}}$$

Here, βgene represents the LASSO regression coefficient of each gene, and Expgene represents gene expression level in each sample.

After the risk score was obtained, we considered the median value as the critical value and divided the samples into HighRisk and LowRisk groups for subsequent analysis.

Furthermore, to validate whether the model has diagnostic value, GSE5281 was utilized. Firstly, the expression values of the model genes were extracted from each sample. Subsequently, in conjunction with the LASSO regression coefficients obtained earlier, the same formula was employed for model construction. ROC curves were then plotted by combining the sample grouping information.

### Comparison of immune microenvironment between HighRisk group and LowRisk group

We downloaded 17 immune gene sets from the Import database (https://www.immport.org/home) [30] using the R package GSVA [31] (version: 1.36.2, http://bioconductor.org/packages/release/bioc/html/GSVA.html), which is based on single-sample enrichment analysis (ssGSEA). We obtained significant differential *P* values between the high- and low-risk groups using the Wilcoxon test. The Spearman correlation coefficients of each gene and the differential immune gene set in the model were calculated, and a correlation heat map was drawn.

In order to understand AD-like group and normal-like immune cells infiltrating differences between groups, here we use the R package MCPcounter (https://github.com/ebecht/MCPcounter) [32] algorithm based on the analysis of the expression level in the data set. The infiltration levels of eight types of immune cells (T cells, CD8 + T cells, cytotoxic lymphocytes, B lineage, NK cells, monocyte lineage, myeloid dendritic cells, and neutrophils) and two types of stromal cells (endothelial cells and fibroblasts) were calculated. Wilcoxon tests were used to compute the significance of differences in the infiltration levels of various immune or stromal cells between the HighRisk and LowRisk groups. Furthermore, we calculated Spearman correlation coefficients between individual genes in the model and differential immune cell infiltrations, and a correlation heatmap was generated to visualize these relationships.

In addition, the Wilcoxon test was used to compare differences in HLA family genes between the AD-like group and normal-like groups. The Pearson correlation coefficient between each gene and the HLA family gene in the model was calculated to construct a correlation heat map.

### Pathway enrichment analysis of the HighRisk and LowRisk groups

A total of 51 *HALLMARK* gene sets were downloaded from MSigDB v7.1 [33] (http://software.broadinstitute.org/gsea/msigdb/index.jsp), and these genes were used as the background for enrichment analysis. The enrichment scores of each HALLMARK pathway in each sample were calculated based on the expression matrix of the analysis data set by the GSVA (gene set variation analysis) algorithm. A scoring matrix was obtained using the GSVA R-packet. Then, a difference analysis of the HighRisk group vs. the LowRisk group was carried out for each pathway using the R packet limma. The results were considered as significant pathways after screening “BH” adjusted (adj. *P* value < 0.05 and |t value|> 2).

### GO BP and KEGG enrichment analysis of key model genes

The key model (in process) and KEGG pathway enrichment analyses were performed using the Cytoscape Software (version 3.4.0, http://chianti.ucsd.edu/cytoscape-3.4.0/) [34] with the Cluego plugin [35] (version 2.5.9, https://apps.cytoscape.org/apps/cluego). The significance threshold was set at a *P* value < 0.05. The Cytoscape software was used to visualize the enrichment results.

## Results

### Data preprocessing and difference analysis

**Fig. 1:**
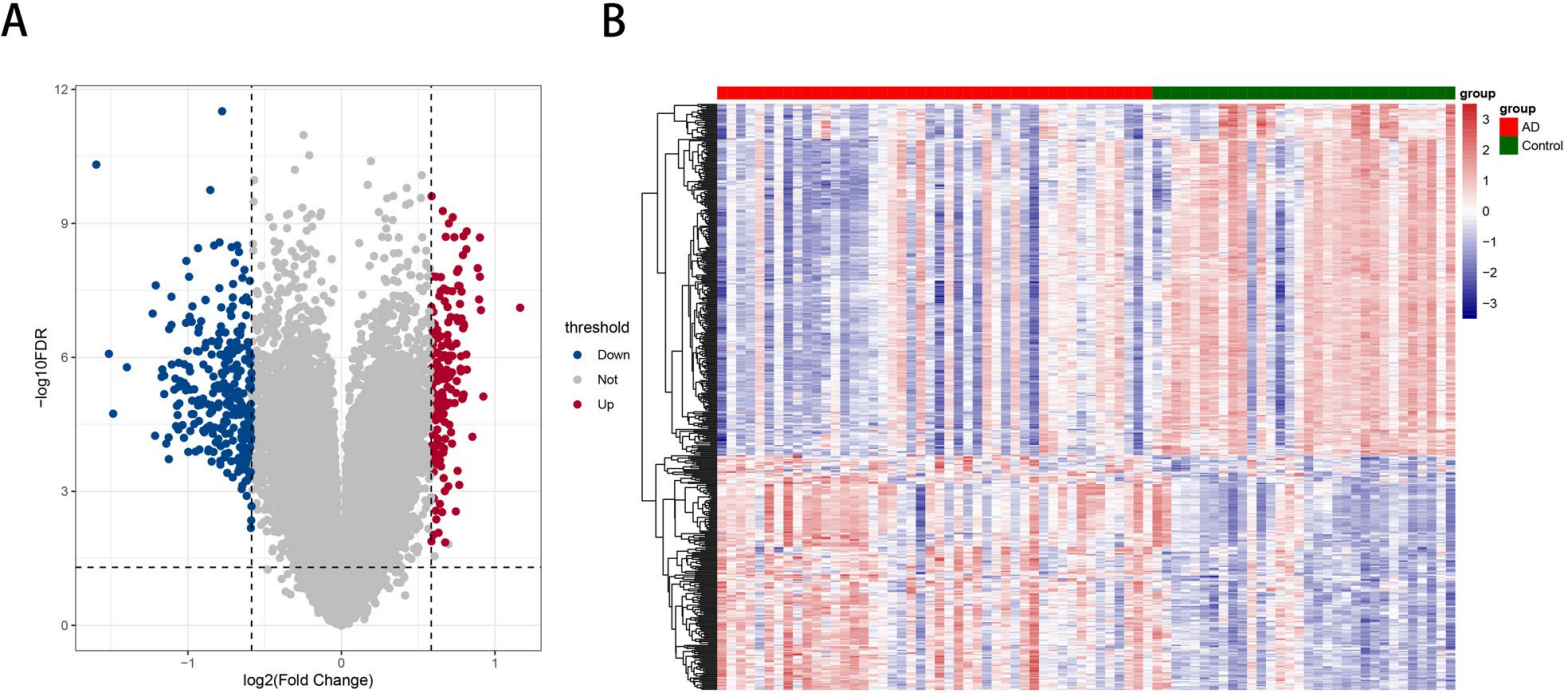
Data preprocessing and Difference analysis. **A** The differential volcanic map; **B** the thermal map

Using the above method, two sets of data were downloaded, preprocessed, and annotated. According to the threshold value, 187 upregulated and 280 downregulated genes were identified. For more details, see Supplementary Table 1. Differential volcanic and thermal maps are shown in Fig. 1A and B.

### WGCNA (Weighted Gene Co-expression Network analysis) was used to analyze disease-associated complement system related gene modules

**Fig. 2:**
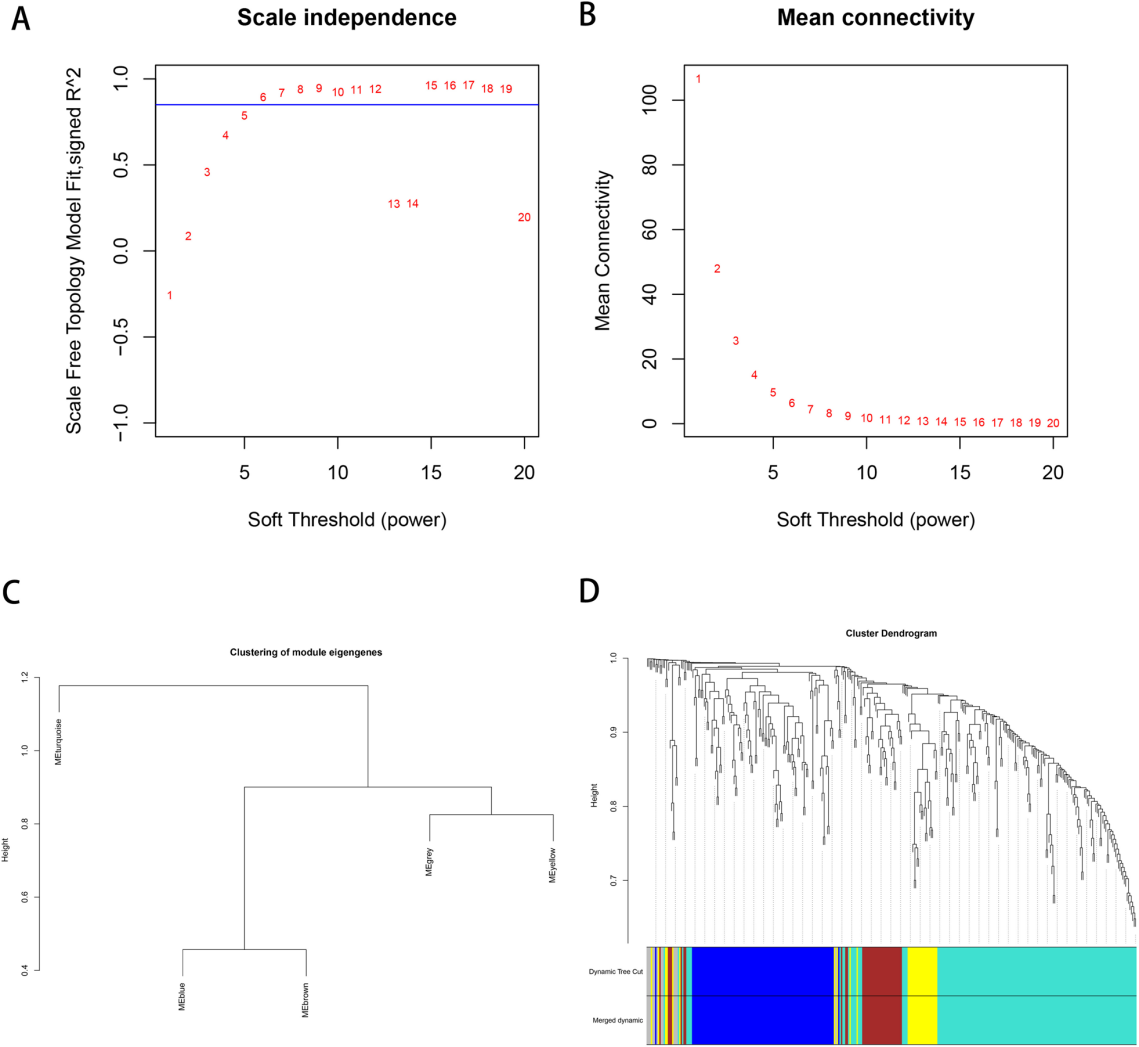
WGCNA of disease-associated complement system related gene. **A** The relationship between scale-free topology model fit and soft-threshold power; **B** The relationship between mean connectivity and soft-threshold power; **C** Clustering of module eigengences; **D** Cluster of dendrogram

Based on a previously described method, first a total of 760 gene expression matrices related to the complementation system were matched. We then chose six as the soft threshold in the WGCNA analysis according to Fig. 2A and B. Second, based on clustering and dynamic pruning methods- high-correlation genes were aggregated into modules; which were then clustered. The modules with correlation coefficients greater than 0.75, that is, the modules with dissimilarity coefficients less than 0.25; were merged and finally integrated into four modules (gray indicates genes with less than any enriched module) (Fig. 2C and D).

**Fig. 3:**
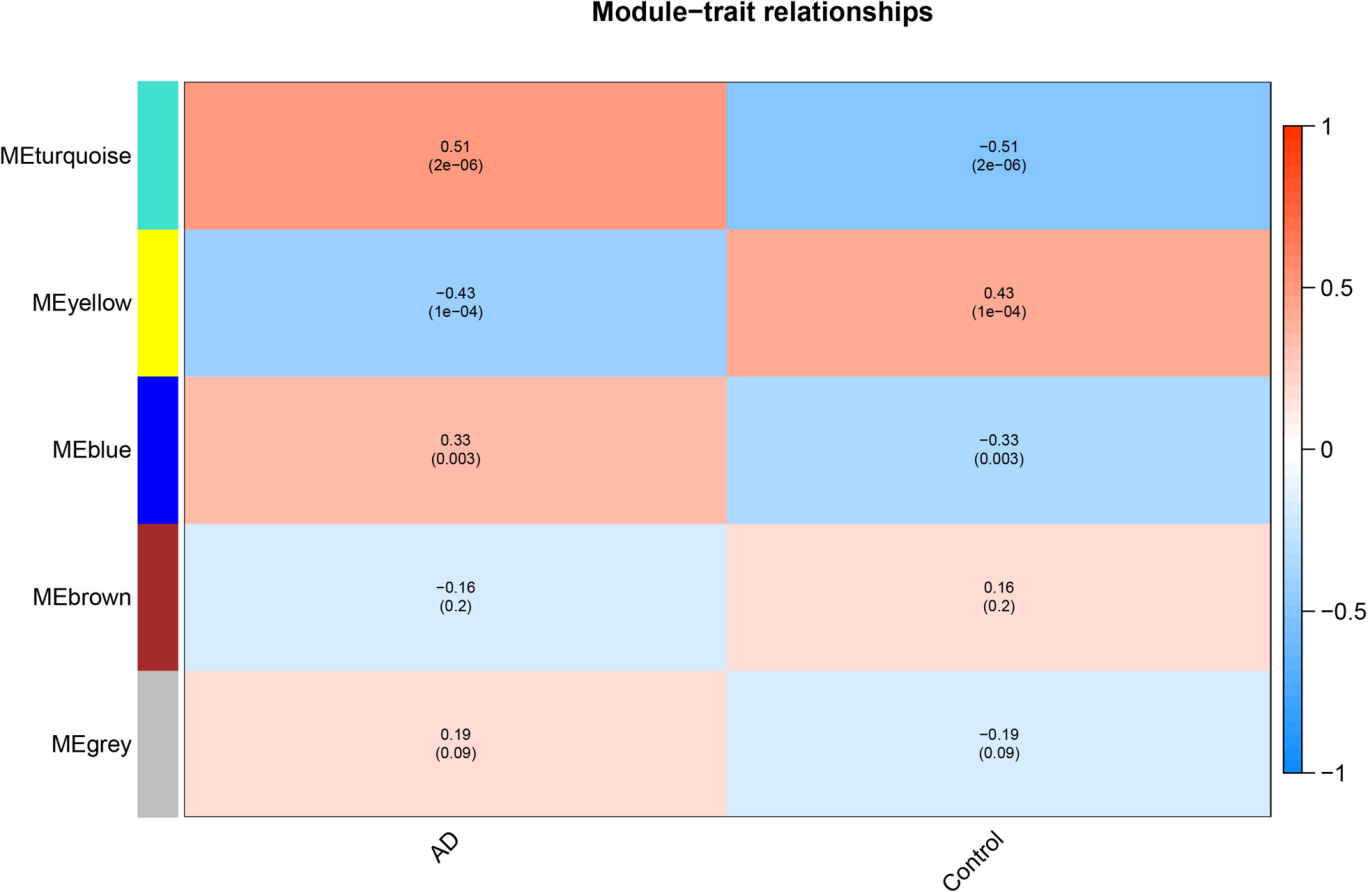
The correlation between the feature vector gene of each module

Furthermore, by calculating the correlation between the feature vector gene of each module (the feature vector gene is the first principal component gene E of a specific module, representing the overall level of gene expression in the module) and the phenotype (whether the sample is OA or Normal), as shown in Fig. 3, it can be seen that the turquoise module (162 genes; correlation coefficient *r* = 0.51 and *P* Value < 0.01) had the most significant positive correlation with AD; and the blue module (102 genes; correlation coefficient *r* = 0.33, *P* Value = 0.003) also showed a significant positive correlation with AD. The yellow module (31 genes; correlation coefficient *r* = 0.43 and *P* < 0.01) was significantly negatively correlated with AD.

### Acquisition of disease-related differential complement system genes

**Fig. 4:**
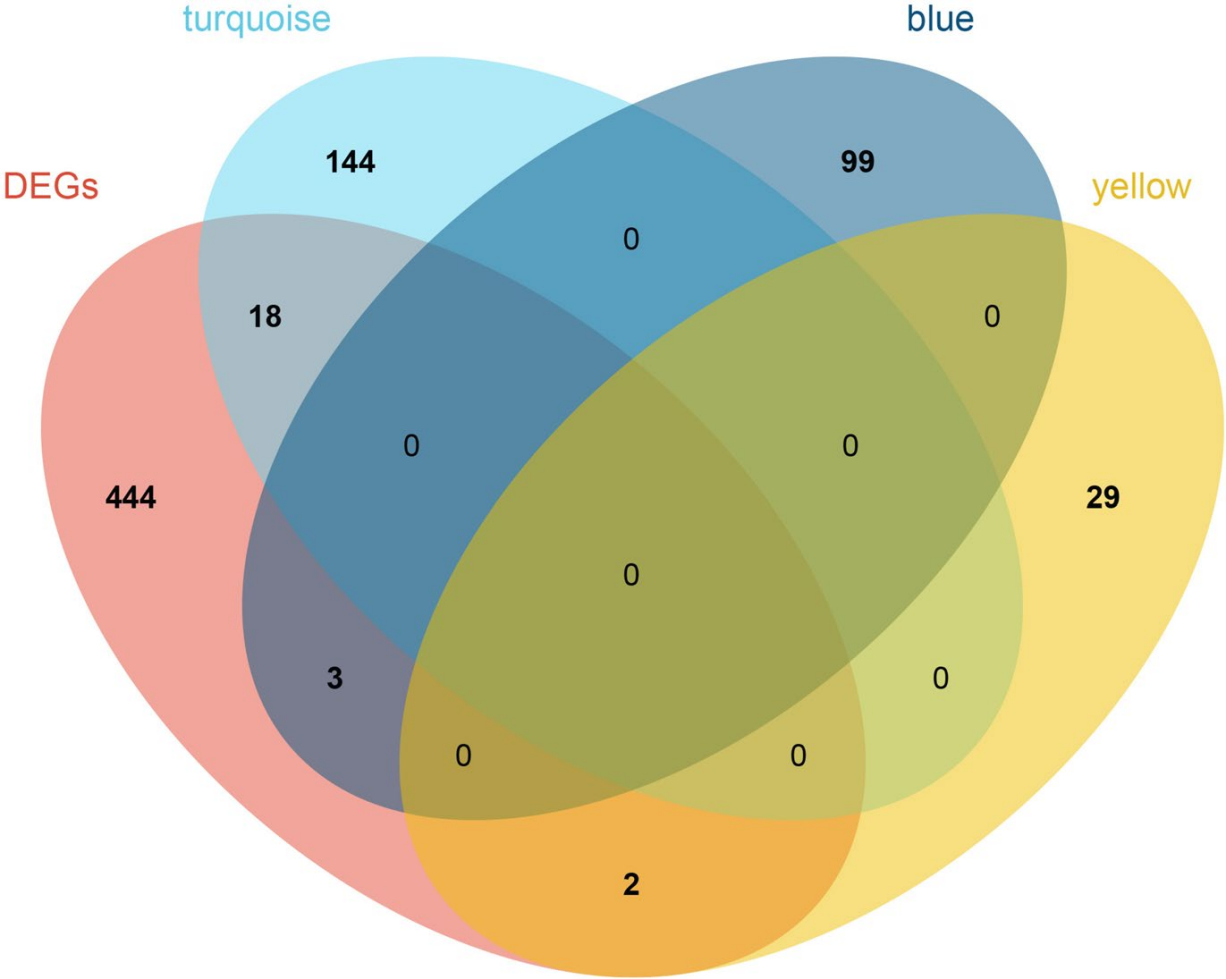
A total of 23 genes were confirmed closely related differential complement system genes of diseases after intersected of disease-related complement system module genes and differential genes

A total of 23 genes were confirmed to be closely related to the differential complement system genes of diseases after intersecting the disease-related complement system module genes and differential genes. As shown in Fig. 4, 18 genes (*GFAP*, *PDGFRB*, *NFKBIA*, *TNFRSF1B*, *NOTCH1*, *BCL6*, *CSF1R*, *LEP*, *DCLRE1C*, *KCNJ10*, *MP2K1*, *VIP*, *SNCA*, *ENO2*, *SST*, *UCHL1*, *HPRT1*, and *STAT4*) belonged to the turquoise module; three genes (*CD14*, *ITGB2*, and *SPP1*) belonged to the blue module; and two genes (*STX1A* and *SYP*) belonged to the yellow module. These three modules were deemed as pivotal module genes associated with AD.

### Protein interaction network and correlation analysis

**Fig. 5:**
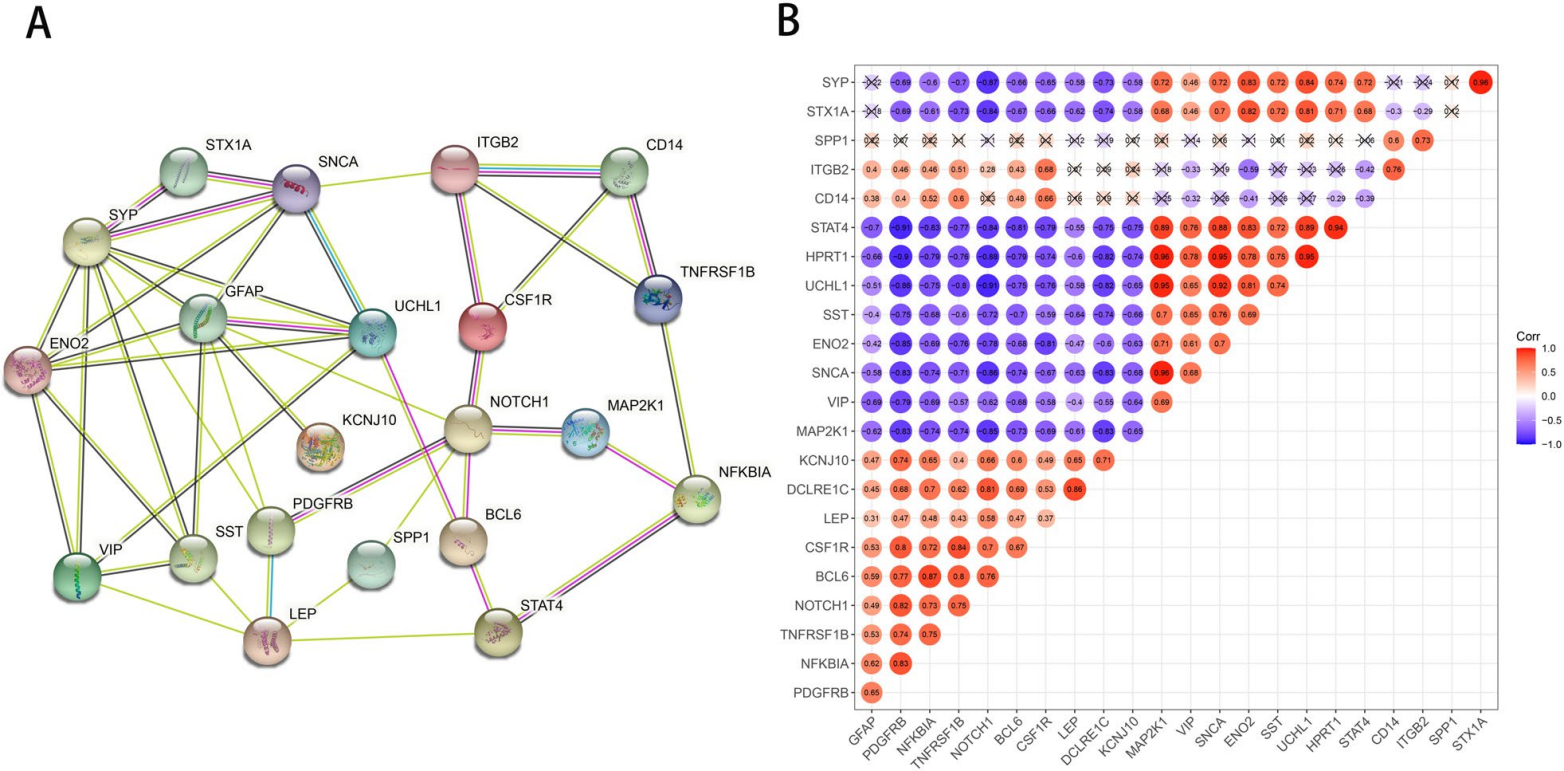
Protein interaction network and correlation analysis. **A** PPI network. There were 21 genes; **B** the Pearson correlation coefficient between the gene and significant *P* value values in the 23 differences of the complement system

The STRING database was used to predict the interactions between the modular gene proteins of the differential complement system. As shown in Fig. 5A, 21 genes and 44 interaction pairs showed close interactions. We further calculated the Pearson correlation coefficient between the genes and significant *P*-values for the 23 differences in the complement system. Except for *SPP1*, the other genes had close and significant positive or negative relationships (Fig. 5B). The results of PPI analysis suggest potential interactions between the complement system and various related proteins, providing new directions for our future exploration of the role of the complement system in AD.

### Key gene screening and diagnostic model construction

Using the method described above, 23 genes related to the complement system were identified. The diagnostic AUC value was calculated by combining it with the sample grouping. Eleven genes with an AUC > 0.8 were obtained (which were initially used as disease-related candidate genes) and were identified.

**Fig. 6:**
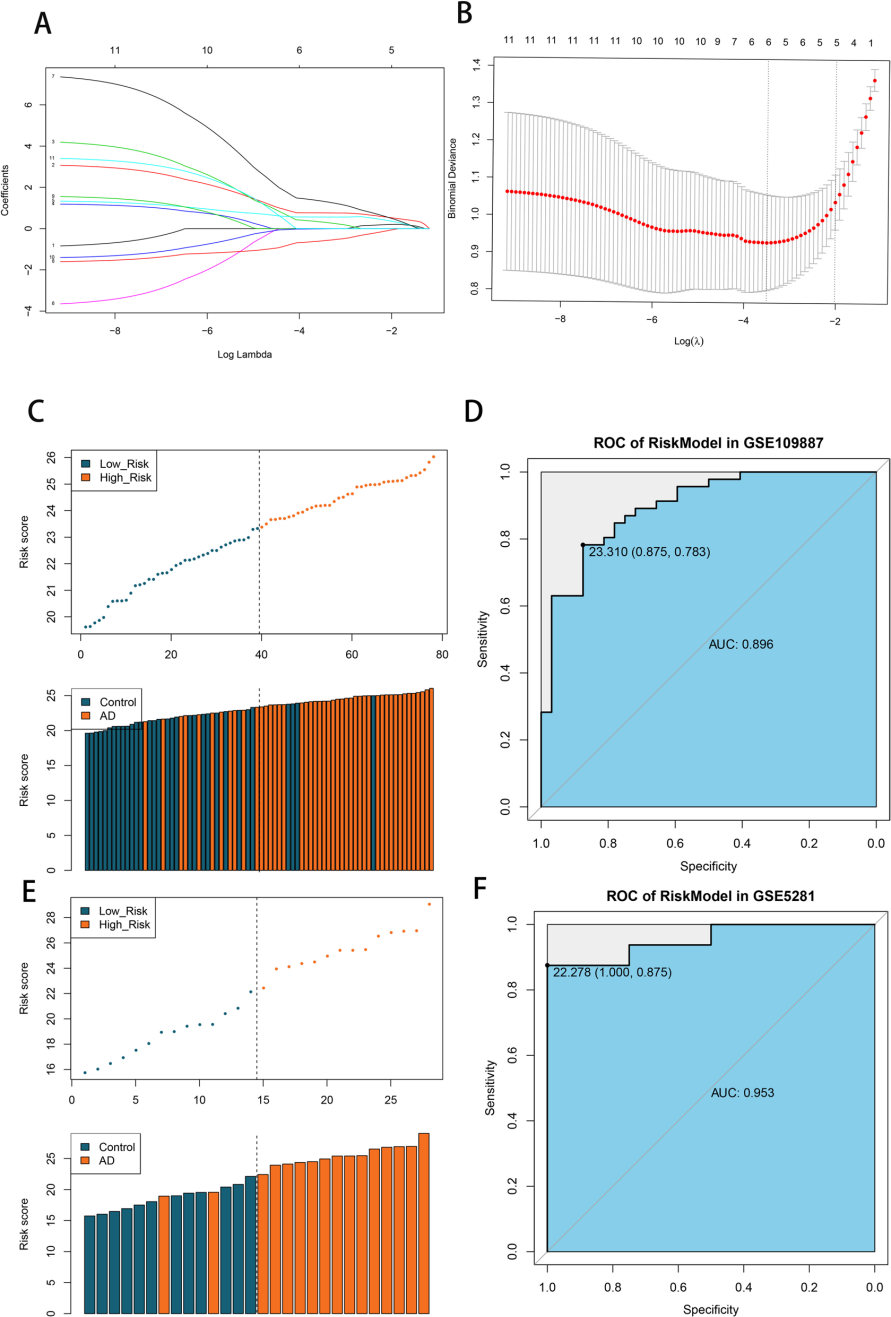
Key gene screening and diagnostic model construction. **A**-**B** Six key characteristic genes *NFKBIA*, *TNFRSF1B*, *BCL6*, *KCNJ10*, *VIP* and *SST* were obtained. **C**-**D** RiskScore model was constructed by combining the above 6 genes and corresponding regression coefficients. **E**-**F** The prediction effect of the model

Further, LASSO regression was performed for these 11 genes- and the results are shown in Fig. 6A-B. Six key characteristic genes, namely *NFKBIA*, *TNFRSF1B*, *BCL6*, *KCNJ10*, *VIP*, and *SST*, were identified.

According to the method described above, the RiskScore model was constructed by combining the above six genes and their corresponding regression coefficients. The results are shown in Fig. 6C-D. According to the ROC curve, the area under the curve exceeded 0.85: indicating that this model had a good disease prediction effect.

To verify the prediction effect of the model, it was reconstructed in GSE5281 according to this same method- and the results are shown in Fig. 6E-F. The area under the curve (AUC) is above 0.9, which is consistent with previous conclusions, indicating that the prediction effect of the model is good.

### Comparison of immune microenvironment between HighRisk group and LowRisk group

**Fig. 7:**
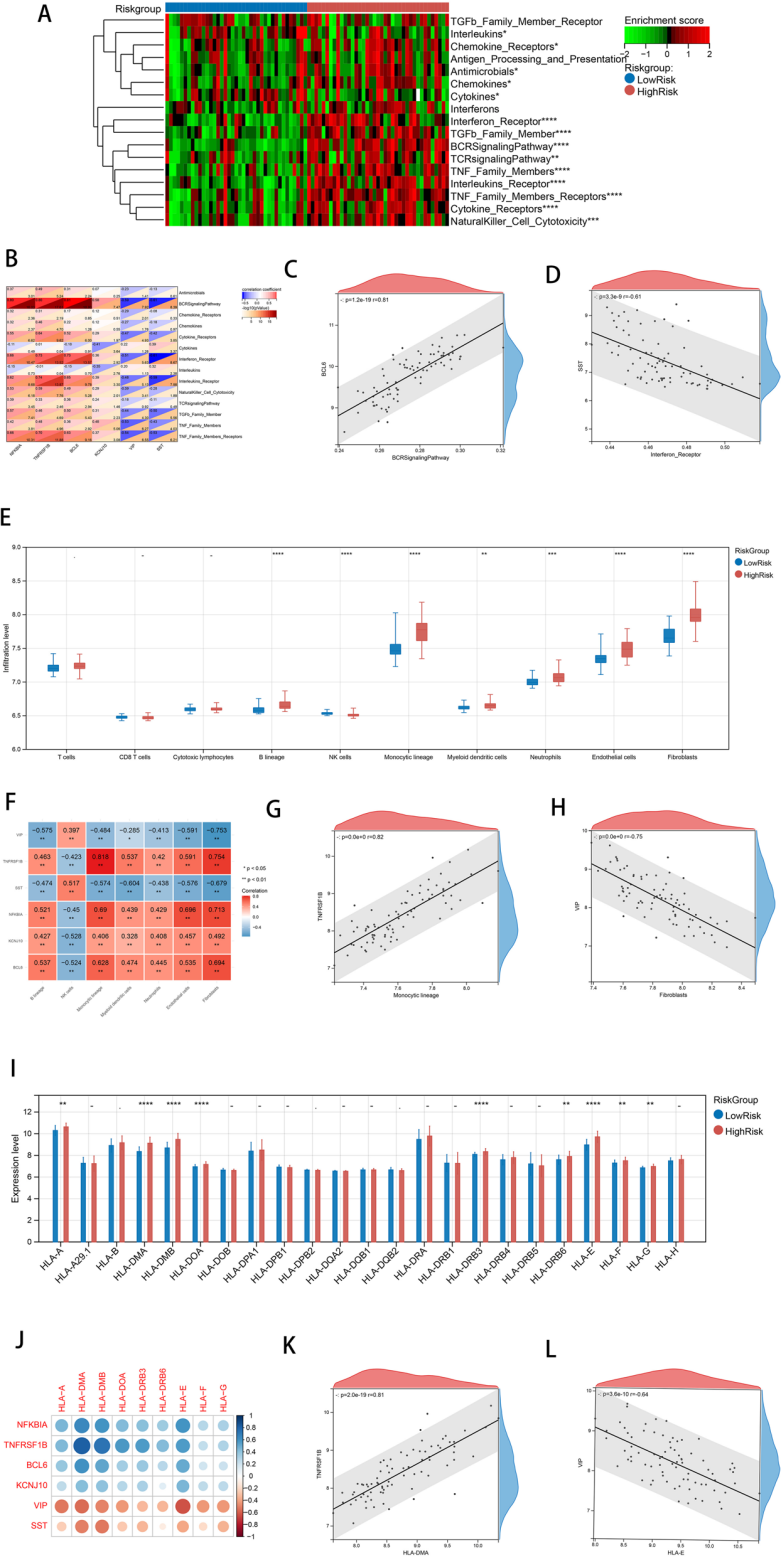
Comparison of immune microenvironment between HighRisk group and LowRisk group. **A** The results showed that a total of 14 immune gene sets showed significant differences between high and low risk groups. **B** The correlation heat map between model genes and 14 different immune gene sets was drawn. **C**-**D** The relationship with the largest positive and negative correlation coefficients was selected for display; **E** 7 kinds of immune cells showed significant differences; **F** The heat map of the correlation between model genes and differential immune cells; **G**-**H** The relationship with the largest positive and negative correlation coefficients was selected for display; **I** The differences of HLA family genes between the HighRisk group and the LowRisk group; **J** 9 genes showed significant differences. **J** The correlation heat map of model genes and different HLA family genes; **K**-**L** The relationship with the largest positive correlation coefficient and negative correlation coefficient was selected to display

According to the method described above, 17 types of immune gene sets were analyzed in accordance with the method described for the HighRisk and LowRisk group comparisons. The results showed that a total of 14 immune gene sets showed significant differences between high and low-risk groups, as shown in Fig. 7A. For this purpose, a correlation heat map was drawn between the model genes and the 14 different immune gene sets as shown in Fig. 7B. The relationships with the largest positive and negative correlation coefficients were selected for display, as shown in Fig. 7C-D.

Furthermore, the immune and stromal cells in the high- and low-risk groups were compared. As shown in Fig. 7E, seven types of immune cells showed significant differences; except for NK cells in the high-risk group, which were lower than those in the low-risk group. The other 6 kinds of cells were significantly higher in the high-risk group than in the low-risk group. A heat map of the correlation between the model genes and differentially expressed immune cells is shown in Fig. 7F. The relationships with the largest positive and negative correlation coefficients were selected for display, as shown in Fig. 7G-H.

According to the method described above, we also compared the differences in HLA family genes between the high- and low-risk groups, as shown in Fig. 7I. Fifteen and nine genes showed significant differences, respectively. A correlation heat map of the model genes and different HLA family genes was drawn, as shown in Fig. 7J. The relationship with the largest positive and negative correlation coefficients was selected for display, as shown in Fig. 7K-L. The results of this section suggest that the complement system may be involved in the pathological mechanisms of AD through interactions with immune-related molecules. This points towards future research directions of AD.

### Pathway enrichment analysis in high-risk and low-risk groups

**Fig. 8:**
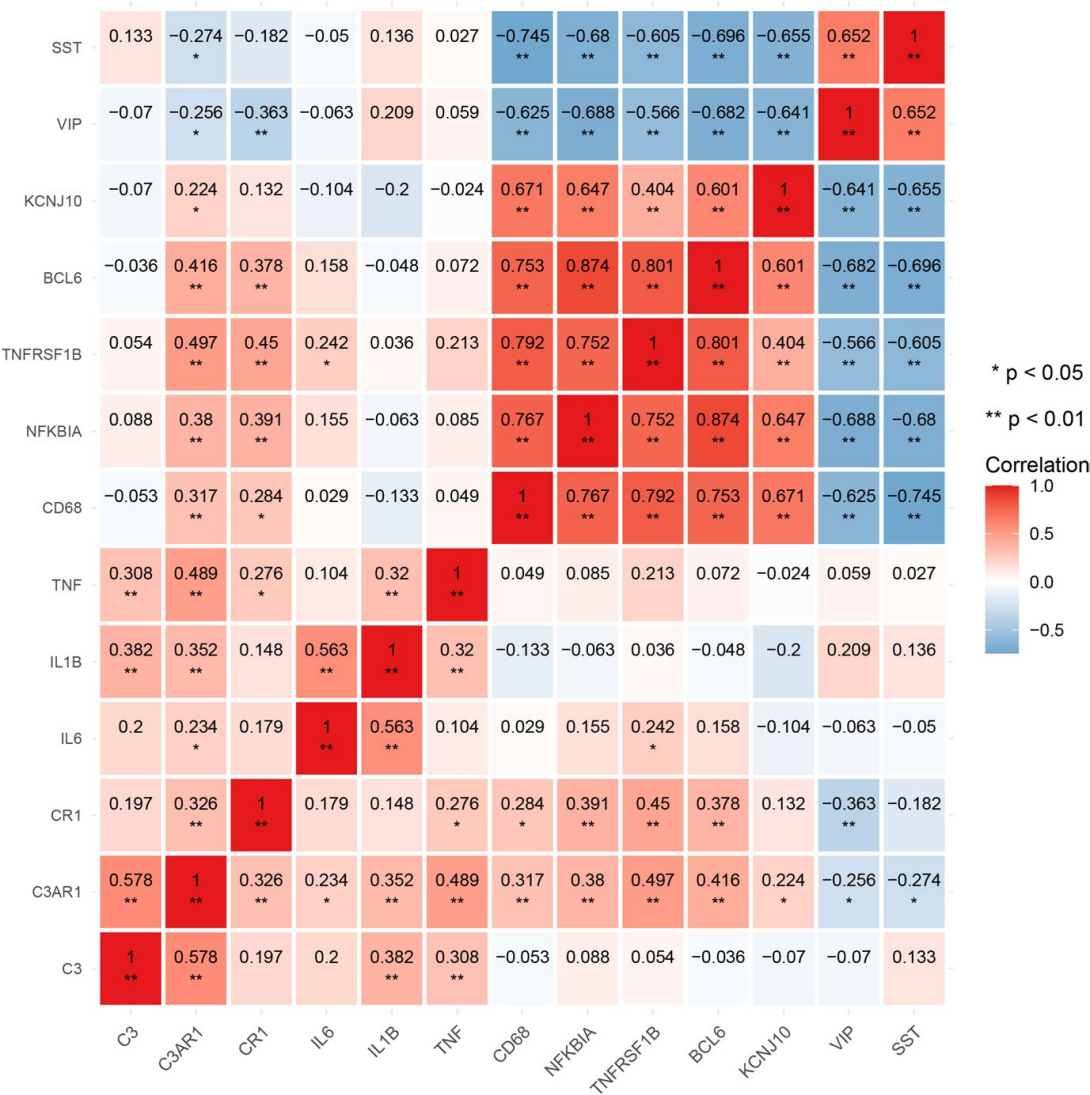
Pathway enrichment analysis in high-risk and low-risk groups

The enrichment scores of each HALLMARK path in each sample were calculated using the GSVA algorithm. According to the method threshold, 15 HALLMARK pathways in HighRisk and 19 low-risk HALLMARK pathways were significantly enriched; as shown in Fig. 8.

### GO BP and KEGG enrichment analysis of key model genes

**Fig. 9:**
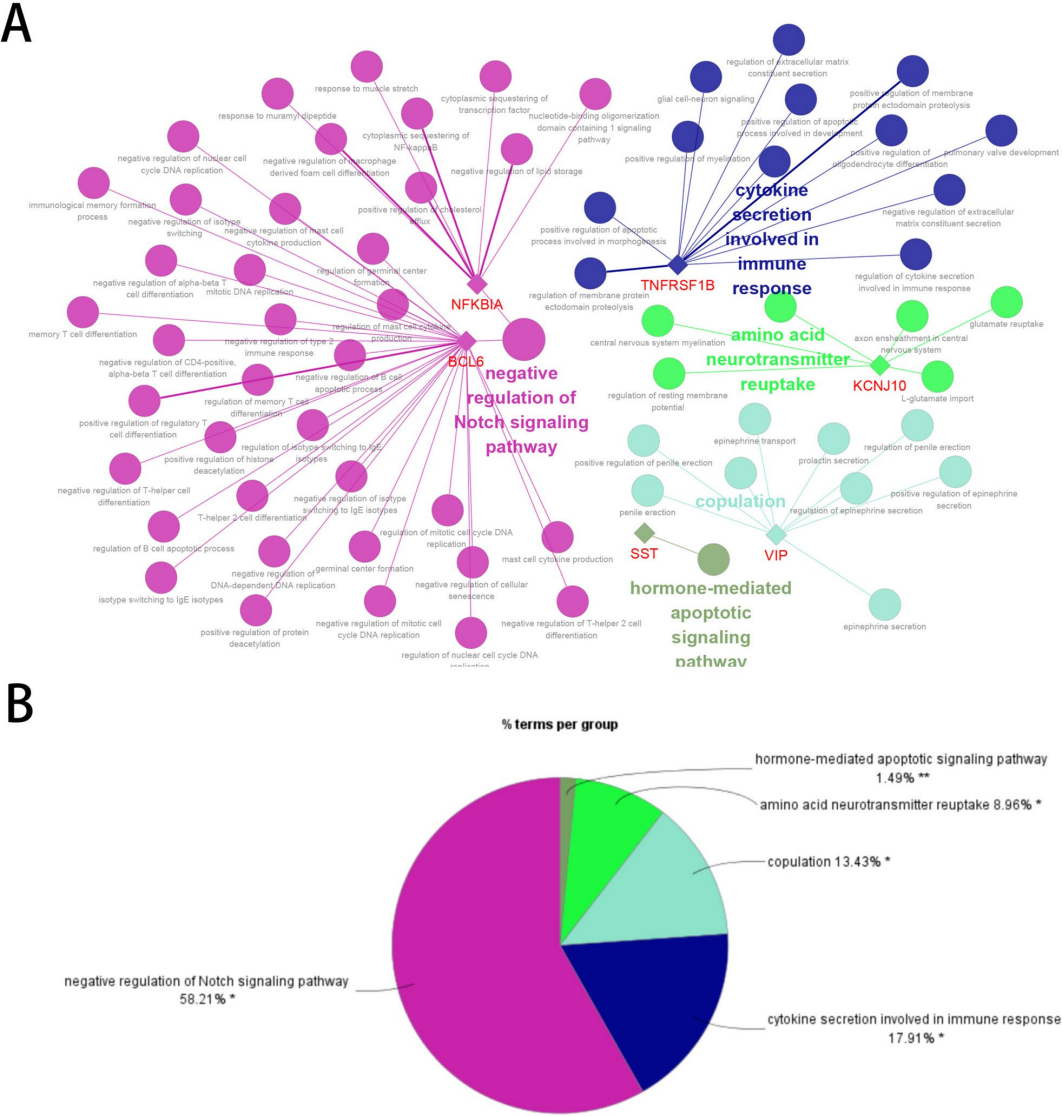
GO BP and KEGG enrichment analysis of key model genes. **A** A total of 67 GO BP; **B** Other key functions

A total of 67 GO BP were enriched in Fig. 9A); however, no KEGG pathways were enriched. Mainly enriched negative regulation of Notch signaling, cytokine secretion involved in the immune response, hormone-mediated apoptotic signaling pathway, and other key functions are shown in Fig. 9B. These studies suggest that the complement system may be involved in the pathological processes of AD through the above signaling pathways. However, the specific mechanisms require further validation.

## Discussion

In this study, the gene information related to AD was analyzed using biogenic analysis. We also identified differences in the regulation of complement-system-related proteins associated with them. A model was constructed to predict the molecular characteristics of AD. Moreover, the relationship between the complement system and AD affecting immune-related genes and inflammatory factors is discussed. This is the first biogenic analysis to comprehensively explore the roles and possible mechanisms of the complement system in AD.

Late-onset AD (LOAD), which accounts for approximately 95% of AD cases, is a multifactorial disease with a heritability of over 58% [36]. Since 2009, large genome-wide association studies (GWAS) have identified more than 75 independent genetic risk factors for LOAD [37–40]. Several studies have been conducted to explore the link between complement system genes and AD. Among the GWAS statistically significant (GWS) hits are two genes encoding proteins of the complement pathway: *CR1* encoding the membrane protein complement receptor 1 (CR1) and *CLU* encoding the plasma regulator clusterin [38]. *CR1* and *CLU* are among the most significant GWAS hits, ranking high in the top 10. These strong associations provide the impetus for this review of complement genetics in LOAD. Our study identified several complement system-related genes in AD. Among the 23 genes previous studies have shown their correlation with AD, we have: *GFAP* [41, 42], *PDGFRβ* [43–45], *NFKBIA* [46], *TNFRSF1B* [47], *NOTCH1* [48], *BCL6* [49], *CSF1R* [50], *LEP* [51], *DCLRE1C* [52], *KCNJ10* [53], *MAP2K1* [54], *VIP* [55], *SNCA* [56], *ENO2* [57], *SST* [58], *UCHL1* [59], *HPRT1* [60], *STAT4* [61], *CD14* [62], *ITGB2* [63], *SPP1* [64], *STX1A* [65], and *SYP* [66]. And these genes are involved in the regulation of molecules associated with the complement system, for instance, *GFAP* [67], *PDGFRβ* [68], *NFKBIA* [69], *TNFRSF1B* [70], *NOTCH1* [71], *BCL6* [72], *CSF1R* [73], *LEP* [74], *DCLRE1C* [75], *KCNJ10* [76], *MAP2K1* [77], *VIP* [78], *SNCA* [79], *ENO2* [80], *SST* [81], *UCHL1* [82], *HPRT1* [83], *STAT4* [84], *CD14* [85], *ITGB2* [86], *SPP1* [87], *STX1A* [88], and *SYP* [89]. However, few studies have discussed their role in AD pathology by modulating the complement system, our study may be a new perspective for our future exploration of the relationship between AD and the complement system. A systematic discussion of the relationship among these genes, the complement system, and AD is likely to become one of the directions for future AD research. The above results show that these proteins are interrelated, and further research is needed to understand their mechanisms. Our research revealed enrichment in the negative regulation of the Notch signaling pathway, cytokine secretion associated with the immune response pathway, and the hormone-mediated apoptotic signaling pathway. These signaling pathway may play a vital role in AD pathology in conjunction with the complement system. However, further experiments are required to investigate this hypothesis.

It is well known that AD lacks effective diagnostic measures. Hence, it may be worthwhile looking into potential brain complement specific biomarkers which can indicate the susceptibility of AD. The classic AD biomarkers are core CSF biomarkers: Aß, t-tau, and phosphorylated tau protein (p-tau), and PET imaging of glucose metabolism and amyloid deposition. However, some patients with early-stage AD cannot be diagnosed using these means [90]. Our findings propose that the diagnostic potential for AD could be enhanced through the consideration of a set of six key genes (*NFKBIA*, *TNFRSF1B*, *BCL6*, *KCNJ10*, *VIP*, and *SST*). When we queried recent literature, we found that *NFKBIA* was associated with AD in some articles [91–95]. The relationship between the *TNFRSF1B* variant rs976881 and the levels of soluble TNFR2 in the cerebrospinal fluid influences various markers of AD severity and cognitive domains [47]. It is reportedly achieved by targeting BCL6, which presents a promising avenue to explore for the prevention and treatment of Aβ-induced neuronal damage in AD [96]. Moreover, genetic screening revealed that mutations in *KCNJ10* play a significant role in neurodegenerative disorders such as AD [53]. VIP may significantly boost microglial uptake of fibrillar Aβ42 and this heightened phagocytic function relied on the activation of the Protein kinase C signaling pathway in AD pathology [97]; VIP was also reported to be involved in Aβ accumulation in AD [98]. A study pointed out an Interaction between Aβ and SST [58]. The validation experiments further affirmed the robust prediction accuracy of our model. It suggests that the model might serve as a diagnostic tool for AD by incorporating classic AD biomarkers and screening for these identified key genes.

## Conclusions

A total of 21 gene proteins and 44 interaction pairs showed close interactions. We screened key genes and created a diagnostic model. The predictive effect of the model was constructed using GSE5281 and our study indicated that the predictive effect of the model was good. Our study also showed enriched negative regulation of Notch signaling, cytokine secretion involved in the immune response pathway and hormone-mediated apoptotic signaling pathway. In this study, key complement system-related genes with good diagnostic value were screened through bioinformatics analysis, which provided some clues for a better understanding of the potential molecular mechanisms of AD. The main limitation of our study is that it primarily involves bioinformatics analysis, lacking relevant clinical or further experimental validation. Hence, these findings of our study must be verified in future studies.

### Supplementary Information

**Additional file 1: Supplementary Table 1.** A total of 187 up-regulated genes and 280 down-regulated genes in Alzheimer’s disease. **Supplementary Figure 1.** Quality Control Preprocess of included data.

## Data Availability

All data during the current study are available from the corresponding author on reasonable request.
