# Supplementary material for: Analysis of complement system and its related factors in Alzheimer’s disease
Source: BMC Neurol. 2023 Dec 19;23:446. doi: 10.1186/s12883-023-03503-0 (PMC10729410; doi:10.1186/s12883-023-03503-0)

**Supplementary Table 1**. A total of 187 up-regulated genes and 280 down-regulated genes in Alzheimer’s disease.

| ***Gene*** | ***logFC*** | ***AveExpr*** | ***t*** | ***P.Value*** | ***adj.P.Val*** | ***B*** |
| --- | --- | --- | --- | --- | --- | --- |
| *SLC5A3* | *1.165255* | *8.786209* | *5.939979* | *7.65E-08* | *1.24E-05* | *7.836709* |
| *GFAP* | *0.92558* | *12.14903* | *4.805058* | *7.43E-06* | *0.000208* | *3.491576* |
| *SLC6A12* | *0.909107* | *8.943163* | *5.909171* | *8.71E-08* | *1.37E-05* | *7.713835* |
| *DDIT4L* | *0.902729* | *9.142835* | *6.316713* | *1.56E-08* | *4.48E-06* | *9.356353* |
| *SLC7A2* | *0.902621* | *8.7267* | *6.785466* | *2.07E-09* | *1.43E-06* | *11.28432* |
| *ITPKB* | *0.897411* | *9.70521* | *6.038653* | *5.06E-08* | *9.74E-06* | *8.231755* |
| *PLXNB1* | *0.888494* | *9.380087* | *6.419312* | *1.00E-08* | *3.44E-06* | *9.775167* |
| *FCGBP* | *0.853519* | *8.545997* | *4.246122* | *5.99E-05* | *0.000864* | *1.526902* |
| *DDIT4* | *0.817085* | *10.0437* | *5.155358* | *1.89E-06* | *8.69E-05* | *4.788094* |
| *TEAD2* | *0.816456* | *8.335296* | *6.86097* | *1.49E-09* | *1.24E-06* | *11.598* |
| *GPER* | *0.814913* | *9.146621* | *5.354416* | *8.51E-07* | *5.43E-05* | *5.544131* |
| *HEY2* | *0.814171* | *8.541417* | *6.647125* | *3.77E-09* | *1.99E-06* | *10.71166* |
| *RAXL1* | *0.800184* | *10.75418* | *5.716375* | *1.94E-07* | *2.11E-05* | *6.950232* |
| *ZCCHC24* | *0.79803* | *9.938975* | *5.739341* | *1.77E-07* | *2.01E-05* | *7.040693* |
| *FLJ38717* | *0.796731* | *9.319213* | *4.832528* | *6.68E-06* | *0.000192* | *3.591567* |
| *PDGFRB* | *0.794851* | *9.942256* | *6.803298* | *1.91E-09* | *1.43E-06* | *11.35834* |
| *AHNAK* | *0.793497* | *9.307202* | *5.682542* | *2.23E-07* | *2.34E-05* | *6.817224* |
| *PTH1R* | *0.792069* | *8.665868* | *4.788608* | *7.91E-06* | *0.000217* | *3.431841* |
| *VCAN* | *0.792014* | *9.339136* | *6.57876* | *5.06E-09* | *2.26E-06* | *10.42973* |
| *ACACB* | *0.787669* | *9.332401* | *4.77111* | *8.46E-06* | *0.000227* | *3.36842* |
| *PTRF* | *0.782904* | *9.76864* | *5.807055* | *1.33E-07* | *1.73E-05* | *7.308209* |
| *VIL2* | *0.782405* | *11.32584* | *6.138223* | *3.32E-08* | *7.26E-06* | *8.632604* |
| *TMEM137* | *0.779821* | *9.601372* | *5.328764* | *9.44E-07* | *5.63E-05* | *5.445975* |
| *ITPRIPL2* | *0.778958* | *9.283942* | *5.134406* | *2.05E-06* | *9.09E-05* | *4.709291* |
| *CALD1* | *0.776373* | *8.594528* | *5.169172* | *1.79E-06* | *8.39E-05* | *4.840132* |
| *C15ORF52* | *0.772546* | *9.099011* | *5.985151* | *6.33E-08* | *1.12E-05* | *8.017279* |
| *LRRC32* | *0.77107* | *8.763923* | *6.205227* | *2.50E-08* | *6.24E-06* | *8.90354* |
| *HSPA1A* | *0.768288* | *10.92936* | *3.526421* | *0.000711* | *0.005219* | *-0.77429* |
| *ITSN1* | *0.762471* | *9.250499* | *6.408606* | *1.05E-08* | *3.51E-06* | *9.731375* |
| *HS.143018* | *0.761907* | *10.21249* | *5.803764* | *1.35E-07* | *1.73E-05* | *7.29518* |
| *BGN* | *0.761622* | *11.01774* | *6.208687* | *2.46E-08* | *6.24E-06* | *8.917554* |
| *ZNF786* | *0.758982* | *10.47364* | *5.772371* | *1.54E-07* | *1.86E-05* | *7.171037* |
| *LOC339879* | *0.756909* | *8.182546* | *3.743279* | *0.000347* | *0.003047* | *-0.11117* |
| *CFLAR* | *0.754304* | *9.508722* | *6.376041* | *1.21E-08* | *3.87E-06* | *9.598296* |
| *SYTL4* | *0.752105* | *8.605322* | *5.006889* | *3.39E-06* | *0.000123* | *4.233041* |
| *SIPA1* | *0.75077* | *8.35171* | *5.817015* | *1.28E-07* | *1.71E-05* | *7.34766* |
| *FOXC1* | *0.749985* | *9.381542* | *5.4942* | *4.83E-07* | *3.70E-05* | *6.082603* |
| *LOC648921* | *0.747715* | *10.32168* | *4.847378* | *6.31E-06* | *0.000186* | *3.645745* |
| *APLNR* | *0.745991* | *9.24695* | *3.089279* | *0.002786* | *0.014687* | *-2.02241* |
| *AEBP1* | *0.738035* | *8.464531* | *4.715476* | *1.05E-05* | *0.000262* | *3.167604* |
| *CHST6* | *0.735491* | *7.653939* | *6.790506* | *2.02E-09* | *1.43E-06* | *11.30524* |
| *NFKBIA* | *0.725858* | *10.87412* | *7.025323* | *7.27E-10* | *8.20E-07* | *12.28332* |
| *LOC100130053* | *0.723255* | *10.65483* | *5.334862* | *9.21E-07* | *5.59E-05* | *5.469291* |
| *TRABD* | *0.722381* | *10.02403* | *6.139462* | *3.31E-08* | *7.26E-06* | *8.637605* |
| *SFMBT2* | *0.721346* | *8.125959* | *4.147568* | *8.53E-05* | *0.001105* | *1.195477* |
| *HS.505676* | *0.72134* | *9.756062* | *5.121004* | *2.16E-06* | *9.35E-05* | *4.658966* |
| *FOXO4* | *0.717724* | *10.83899* | *5.454957* | *5.67E-07* | *4.15E-05* | *5.930833* |
| *RUNDC2C* | *0.716574* | *9.086298* | *5.123112* | *2.15E-06* | *9.29E-05* | *4.666878* |
| *HS.37648* | *0.714982* | *8.449101* | *4.309025* | *4.77E-05* | *0.00073* | *1.740924* |
| *LOC643287* | *0.714874* | *9.08342* | *4.89807* | *5.19E-06* | *0.000163* | *3.831332* |
| *RN7SK* | *0.712899* | *9.466032* | *5.822358* | *1.25E-07* | *1.69E-05* | *7.368831* |
| *TMEM106A* | *0.711545* | *9.521659* | *5.119036* | *2.18E-06* | *9.39E-05* | *4.651581* |
| *ZFP36L1* | *0.70894* | *10.03024* | *5.091058* | *2.44E-06* | *0.000101* | *4.546745* |
| *INPPL1* | *0.708135* | *8.989052* | *5.339202* | *9.05E-07* | *5.55E-05* | *5.48589* |
| *CEP27* | *0.70743* | *10.8556* | *5.109008* | *2.27E-06* | *9.66E-05* | *4.613973* |
| *MT2A* | *0.707211* | *11.22836* | *4.716823* | *1.04E-05* | *0.000261* | *3.172452* |
| *TNFSF14* | *0.703867* | *10.13527* | *5.063875* | *2.71E-06* | *0.000107* | *4.44515* |
| *HSPB1* | *0.703339* | *11.87765* | *4.415593* | *3.23E-05* | *0.000565* | *2.107803* |
| *TNFRSF1B* | *0.702508* | *8.417308* | *5.330733* | *9.37E-07* | *5.62E-05* | *5.453504* |
| *RHOQ* | *0.700542* | *10.15687* | *6.951492* | *1.00E-09* | *9.94E-07* | *11.97506* |
| *C10ORF10* | *0.697013* | *8.852442* | *3.502095* | *0.00077* | *0.005536* | *-0.84692* |
| *FLJ25363* | *0.697012* | *10.21254* | *5.4789* | *5.14E-07* | *3.88E-05* | *6.023375* |
| *NOTCH1* | *0.696721* | *9.266233* | *5.758786* | *1.63E-07* | *1.91E-05* | *7.117392* |
| *LOC100129828* | *0.696602* | *8.749972* | *5.272453* | *1.18E-06* | *6.49E-05* | *5.231247* |
| *HLA-DMB* | *0.696413* | *9.082535* | *5.330006* | *9.39E-07* | *5.62E-05* | *5.450723* |
| *RASL12* | *0.693617* | *8.242203* | *5.518504* | *4.38E-07* | *3.50E-05* | *6.176827* |
| *GSDMD* | *0.692724* | *8.018966* | *5.599448* | *3.14E-07* | *2.84E-05* | *6.491882* |
| *ITGB5* | *0.691688* | *8.733212* | *5.949042* | *7.37E-08* | *1.23E-05* | *7.872899* |
| *FZD9* | *0.690755* | *8.954405* | *4.933165* | *4.52E-06* | *0.000151* | *3.960401* |
| *EZR* | *0.689919* | *12.06325* | *6.147827* | *3.19E-08* | *7.26E-06* | *8.671381* |
| *RAPGEF3* | *0.685137* | *8.217813* | *5.15566* | *1.89E-06* | *8.69E-05* | *4.78923* |
| *LOC100133019* | *0.683825* | *8.419559* | *4.726475* | *1.00E-05* | *0.000254* | *3.207204* |
| *ANLN* | *0.683061* | *9.181593* | *5.035539* | *3.03E-06* | *0.000115* | *4.339534* |
| *COX19* | *0.682639* | *9.918125* | *4.676893* | *1.21E-05* | *0.000288* | *3.029083* |
| *BCL6* | *0.680466* | *9.781167* | *6.792951* | *2.00E-09* | *1.43E-06* | *11.31538* |
| *MYOT* | *0.679118* | *8.169879* | *5.109195* | *2.27E-06* | *9.66E-05* | *4.614674* |
| *EML3* | *0.678422* | *9.446114* | *4.704424* | *1.09E-05* | *0.00027* | *3.127861* |
| *DDX27* | *0.676224* | *8.728659* | *4.217896* | *6.63E-05* | *0.000922* | *1.431491* |
| *S100A8* | *0.676151* | *8.435729* | *2.519628* | *0.013807* | *0.049139* | *-3.45188* |
| *LOC642031* | *0.674882* | *9.227325* | *5.343713* | *8.89E-07* | *5.51E-05* | *5.503151* |
| *HS.534061* | *0.674741* | *9.898161* | *3.427288* | *0.000979* | *0.006627* | *-1.06801* |
| *FAM107B* | *0.673449* | *9.618637* | *4.455321* | *2.78E-05* | *0.000506* | *2.245922* |
| *CCDC125* | *0.673235* | *11.6203* | *6.018445* | *5.51E-08* | *1.01E-05* | *8.150671* |
| *NUBPL* | *0.67247* | *10.60062* | *5.075698* | *2.59E-06* | *0.000104* | *4.489305* |
| *DLC1* | *0.668458* | *9.092429* | *4.077634* | *0.000109* | *0.001307* | *0.963255* |
| *ADAMTS1* | *0.667808* | *8.204142* | *5.125126* | *2.13E-06* | *9.25E-05* | *4.674438* |
| *ITPRIP* | *0.667424* | *8.055081* | *6.175928* | *2.83E-08* | *6.65E-06* | *8.784953* |
| *C9ORF130* | *0.667111* | *9.831605* | *4.571022* | *1.81E-05* | *0.000382* | *2.652207* |
| *BCAS1* | *0.667063* | *10.31102* | *3.632825* | *0.000502* | *0.004028* | *-0.45239* |
| *SYNJ2BP* | *0.665633* | *9.958693* | *5.04751* | *2.89E-06* | *0.000112* | *4.384118* |
| *CSF1R* | *0.664733* | *10.10408* | *5.036234* | *3.02E-06* | *0.000115* | *4.34212* |
| *MT1M* | *0.663996* | *9.163728* | *4.018881* | *0.000135* | *0.001522* | *0.770108* |
| *EIF2AK4* | *0.661149* | *9.890873* | *4.764083* | *8.69E-06* | *0.000231* | *3.342986* |
| *MLLT6* | *0.66045* | *9.817661* | *7.09908* | *5.27E-10* | *8.10E-07* | *12.59191* |
| *RAB13* | *0.659793* | *7.858097* | *4.810706* | *7.27E-06* | *0.000205* | *3.512109* |
| *HLA-DMA* | *0.657658* | *8.740431* | *5.316945* | *9.90E-07* | *5.75E-05* | *5.400821* |
| *MT1H* | *0.656779* | *8.71686* | *3.07385* | *0.002918* | *0.015183* | *-2.06417* |
| *ZNF223* | *0.656659* | *10.85678* | *4.859931* | *6.01E-06* | *0.00018* | *3.69161* |
| *SLC25A18* | *0.655497* | *10.23944* | *4.663335* | *1.28E-05* | *0.000299* | *2.980555* |
| *AXUD1* | *0.654781* | *9.370206* | *5.462339* | *5.50E-07* | *4.06E-05* | *5.959344* |
| *SLC16A9* | *0.653032* | *9.660411* | *4.111038* | *9.72E-05* | *0.001212* | *1.073865* |
| *CGNL1* | *0.651499* | *10.7126* | *5.135139* | *2.05E-06* | *9.09E-05* | *4.712046* |
| *LOC100133692* | *0.650921* | *8.630127* | *4.604596* | *1.59E-05* | *0.000348* | *2.7712* |
| *CLDN5* | *0.649135* | *10.10972* | *3.811265* | *0.000276* | *0.002569* | *0.102329* |
| *CENPB* | *0.64896* | *10.4071* | *4.818326* | *7.06E-06* | *0.0002* | *3.539833* |
| *PPFIBP2* | *0.648156* | *8.819435* | *6.312772* | *1.58E-08* | *4.48E-06* | *9.340305* |
| *SLC15A3* | *0.647953* | *8.528121* | *5.581998* | *3.37E-07* | *3.00E-05* | *6.423803* |
| *CUEDC1* | *0.647836* | *9.487584* | *4.505865* | *2.31E-05* | *0.000448* | *2.422674* |
| *LOC284988* | *0.647415* | *9.248526* | *6.139113* | *3.31E-08* | *7.26E-06* | *8.636195* |
| *LOC389765* | *0.645613* | *10.02865* | *4.746252* | *9.31E-06* | *0.000242* | *3.278536* |
| *LOC644250* | *0.644187* | *11.1636* | *5.029219* | *3.11E-06* | *0.000117* | *4.316016* |
| *LOC649362* | *0.643273* | *7.679165* | *3.905552* | *0.0002* | *0.002023* | *0.402695* |
| *FXYD5* | *0.642621* | *8.996836* | *5.042373* | *2.95E-06* | *0.000113* | *4.364977* |
| *KIAA1881* | *0.642316* | *8.22473* | *5.09911* | *2.36E-06* | *9.89E-05* | *4.576888* |
| *MT1F* | *0.641485* | *11.59462* | *3.213743* | *0.001911* | *0.011019* | *-1.67967* |
| *PLOD1* | *0.641364* | *10.10164* | *5.358442* | *8.38E-07* | *5.39E-05* | *5.559557* |
| *USP49* | *0.639845* | *10.96077* | *4.794535* | *7.74E-06* | *0.000214* | *3.453352* |
| *LASS1* | *0.638383* | *8.672269* | *6.080853* | *4.23E-08* | *8.62E-06* | *8.401379* |
| *ZBTB20* | *0.637435* | *9.791098* | *5.194242* | *1.62E-06* | *7.96E-05* | *4.93474* |
| *LOC100129681* | *0.636756* | *8.776097* | *3.987861* | *0.00015* | *0.001647* | *0.66886* |
| *AKR1C3* | *0.636484* | *9.471537* | *4.615589* | *1.53E-05* | *0.000339* | *2.810269* |
| *RGS1* | *0.633523* | *7.871727* | *2.702175* | *0.008461* | *0.033837* | *-3.01964* |
| *SYAP1* | *0.633392* | *9.869219* | *5.085836* | *2.49E-06* | *0.000102* | *4.527208* |
| *CD68* | *0.631157* | *9.740952* | *5.561534* | *3.67E-07* | *3.16E-05* | *6.344074* |
| *NDE1* | *0.6311* | *8.667765* | *5.558696* | *3.71E-07* | *3.17E-05* | *6.333028* |
| *UBXN2A* | *0.630256* | *9.114687* | *4.820052* | *7.01E-06* | *0.000199* | *3.546119* |
| *HS.397465* | *0.630002* | *9.732839* | *4.155949* | *8.28E-05* | *0.001081* | *1.223472* |
| *LEP* | *0.629595* | *8.684907* | *4.756978* | *8.93E-06* | *0.000237* | *3.31729* |
| *TMEM191A* | *0.627165* | *9.282419* | *4.195278* | *7.19E-05* | *0.000977* | *1.355319* |
| *LYL1* | *0.62666* | *8.246852* | *5.008117* | *3.38E-06* | *0.000123* | *4.237597* |
| *CD14* | *0.626243* | *9.969114* | *3.843281* | *0.000247* | *0.002376* | *0.20377* |
| *LPAR1* | *0.625733* | *9.79616* | *4.227065* | *6.42E-05* | *0.000902* | *1.462441* |
| *CD151* | *0.625075* | *9.688277* | *5.665395* | *2.40E-07* | *2.43E-05* | *6.74993* |
| *LOC100130598* | *0.624771* | *9.061473* | *4.351482* | *4.08E-05* | *0.000658* | *1.886451* |
| *APOLD1* | *0.623302* | *9.684728* | *4.003504* | *0.000142* | *0.001583* | *0.719854* |
| *CEBPD* | *0.622917* | *10.66968* | *4.54928* | *1.96E-05* | *0.000402* | *2.575406* |
| *LOC400446* | *0.622523* | *11.30271* | *5.255379* | *1.27E-06* | *6.76E-05* | *5.166345* |
| *FAM40B* | *0.621969* | *9.011234* | *4.497484* | *2.38E-05* | *0.000457* | *2.393287* |
| *PDCD4* | *0.621059* | *10.14638* | *5.382172* | *7.61E-07* | *5.05E-05* | *5.650574* |
| *RNU1G2* | *0.620655* | *8.29993* | *4.508643* | *2.28E-05* | *0.000445* | *2.432423* |
| *IL17RB* | *0.620066* | *8.351383* | *5.379542* | *7.69E-07* | *5.09E-05* | *5.640477* |
| *PIGX* | *0.619634* | *8.932227* | *5.149453* | *1.93E-06* | *8.80E-05* | *4.765869* |
| *TNS3* | *0.619302* | *11.24516* | *5.025114* | *3.16E-06* | *0.000118* | *4.300748* |
| *FAM65C* | *0.618917* | *7.217988* | *6.315531* | *1.56E-08* | *4.48E-06* | *9.35154* |
| *NBPF8* | *0.61795* | *9.407344* | *2.949893* | *0.004203* | *0.019973* | *-2.39372* |
| *DCLRE1C* | *0.617739* | *8.5969* | *5.06098* | *2.74E-06* | *0.000108* | *4.434349* |
| *LOC727908* | *0.614771* | *8.402427* | *3.098121* | *0.002713* | *0.014394* | *-1.99841* |
| *LOC100131096* | *0.614723* | *8.711934* | *4.477573* | *2.56E-05* | *0.000481* | *2.323595* |
| *KCNK12* | *0.61435* | *10.66457* | *5.57654* | *3.45E-07* | *3.03E-05* | *6.402525* |
| *HS.379253* | *0.613804* | *8.827412* | *4.333309* | *4.36E-05* | *0.000688* | *1.824058* |
| *ZNF621* | *0.613429* | *8.570958* | *5.625746* | *2.82E-07* | *2.65E-05* | *6.594641* |
| *ALS2CR14* | *0.613239* | *8.943829* | *4.576453* | *1.77E-05* | *0.000377* | *2.671422* |
| *PLA2G2D* | *0.613098* | *8.549101* | *4.604567* | *1.59E-05* | *0.000348* | *2.771098* |
| *PTPLAD2* | *0.612343* | *10.96153* | *4.520948* | *2.18E-05* | *0.000433* | *2.475641* |
| *PALLD* | *0.609638* | *9.808889* | *4.703009* | *1.10E-05* | *0.000271* | *3.122779* |
| *PIP5K2B* | *0.608527* | *11.46024* | *5.734118* | *1.80E-07* | *2.03E-05* | *7.020107* |
| *C20ORF127* | *0.608394* | *9.125896* | *4.103035* | *0.0001* | *0.001231* | *1.047314* |
| *LCP1* | *0.605862* | *8.608261* | *4.121461* | *9.37E-05* | *0.001181* | *1.108495* |
| *LOC727948* | *0.603971* | *8.68419* | *4.546788* | *1.98E-05* | *0.000405* | *2.566616* |
| *TXNIP* | *0.602262* | *11.41501* | *3.304885* | *0.001441* | *0.008912* | *-1.4222* |
| *ITGB2* | *0.601175* | *9.614577* | *3.728116* | *0.000365* | *0.003173* | *-0.15843* |
| *GPR4* | *0.601145* | *7.715203* | *5.377906* | *7.74E-07* | *5.11E-05* | *5.634199* |
| *LRCH4* | *0.600574* | *8.336597* | *4.853054* | *6.17E-06* | *0.000184* | *3.666477* |
| *SPP1* | *0.600157* | *10.31019* | *2.666729* | *0.009322* | *0.036414* | *-3.10554* |
| *LOC100131541* | *0.599919* | *7.92603* | *5.745298* | *1.72E-07* | *1.99E-05* | *7.064177* |
| *CAPS* | *0.599895* | *7.703066* | *5.889729* | *9.44E-08* | *1.43E-05* | *7.636411* |
| *HS.576072* | *0.599852* | *8.620023* | *4.727525* | *1.00E-05* | *0.000253* | *3.210989* |
| *KCNJ10* | *0.598417* | *8.737942* | *6.321672* | *1.52E-08* | *4.48E-06* | *9.376552* |
| *TMC6* | *0.598199* | *7.835748* | *5.636829* | *2.69E-07* | *2.60E-05* | *6.638003* |
| *LOC729120* | *0.597604* | *10.41105* | *5.478187* | *5.16E-07* | *3.88E-05* | *6.020619* |
| *MTE* | *0.596922* | *9.325498* | *3.952719* | *0.00017* | *0.001793* | *0.554775* |
| *LLPH* | *0.596737* | *10.11911* | *4.685592* | *1.17E-05* | *0.000283* | *3.060258* |
| *FLNC* | *0.595971* | *8.418915* | *4.278668* | *5.32E-05* | *0.000794* | *1.6374* |
| *KIF1C* | *0.594972* | *7.741957* | *4.695327* | *1.13E-05* | *0.000276* | *3.095186* |
| *DCN* | *0.594282* | *8.957536* | *4.964054* | *4.01E-06* | *0.00014* | *4.074383* |
| *HS.531457* | *0.594059* | *10.3833* | *5.668476* | *2.37E-07* | *2.41E-05* | *6.762017* |
| *FAM129B* | *0.593849* | *8.575357* | *4.991036* | *3.61E-06* | *0.000129* | *4.174242* |
| *KLF2* | *0.593672* | *10.73272* | *3.855659* | *0.000237* | *0.002299* | *0.243141* |
| *ACSS1* | *0.59132* | *9.225949* | *4.100204* | *0.000101* | *0.001237* | *1.03793* |
| *C10ORF116* | *0.590984* | *10.82994* | *3.999395* | *0.000144* | *0.0016* | *0.706447* |
| *RHBDF1* | *0.588548* | *9.099967* | *5.181371* | *1.70E-06* | *8.18E-05* | *4.88614* |
| *ITPR3* | *0.588464* | *8.643014* | *4.933415* | *4.52E-06* | *0.000151* | *3.96132* |
| *CTDSP2* | *0.588406* | *9.75164* | *7.274136* | *2.44E-10* | *5.80E-07* | *13.32655* |
| *CSF2RA* | *0.587724* | *10.80475* | *5.340937* | *8.99E-07* | *5.55E-05* | *5.492531* |
| *LOC649841* | *0.587216* | *9.768328* | *2.537583* | *0.013171* | *0.047348* | *-3.41049* |
| *FLJ46906* | *0.587169* | *8.207129* | *4.117266* | *9.51E-05* | *0.001193* | *1.094551* |
| *TSPO* | *0.586556* | *8.788925* | *5.94749* | *7.42E-08* | *1.23E-05* | *7.866699* |
| *LOC341457* | *-0.5853* | *11.04536* | *-3.17724* | *0.002136* | *0.011991* | *-1.78128* |
| *RTN3* | *-0.58561* | *10.85624* | *-5.11294* | *2.23E-06* | *9.53E-05* | *4.628703* |
| *LOC100129086* | *-0.58632* | *8.64989* | *-4.51694* | *2.21E-05* | *0.000436* | *2.461543* |
| *YWHAH* | *-0.58639* | *13.21565* | *-4.23362* | *6.27E-05* | *0.00089* | *1.484611* |
| *BSG* | *-0.5876* | *8.571936* | *-2.93319* | *0.004411* | *0.020707* | *-2.4373* |
| *ACTR10* | *-0.5877* | *9.020448* | *-5.03736* | *3.01E-06* | *0.000114* | *4.346317* |
| *PFTK1* | *-0.58847* | *8.710116* | *-4.60924* | *1.57E-05* | *0.000344* | *2.787691* |
| *KCNIP4* | *-0.58915* | *8.400161* | *-4.90986* | *4.95E-06* | *0.000159* | *3.874645* |
| *HERC1* | *-0.58917* | *9.304451* | *-4.70097* | *1.11E-05* | *0.000272* | *3.11546* |
| *SGIP1* | *-0.58924* | *9.158323* | *-5.32885* | *9.44E-07* | *5.63E-05* | *5.446305* |
| *FBXO34* | *-0.58988* | *8.854454* | *-3.72248* | *0.000372* | *0.003216* | *-0.17597* |
| *SLITRK5* | *-0.58991* | *8.459153* | *-4.19436* | *7.22E-05* | *0.000979* | *1.352247* |
| *SYNPR* | *-0.59012* | *9.88136* | *-2.79236* | *0.006589* | *0.028021* | *-2.79693* |
| *TM2D3* | *-0.59071* | *9.425457* | *-4.94429* | *4.33E-06* | *0.000147* | *4.001398* |
| *CNTN3* | *-0.59147* | *7.841435* | *-4.31928* | *4.59E-05* | *0.000711* | *1.775994* |
| *BTBD10* | *-0.59348* | *8.933375* | *-4.35943* | *3.97E-05* | *0.000645* | *1.913773* |
| *NCDN* | *-0.59395* | *9.115186* | *-4.09152* | *0.000104* | *0.001261* | *1.00918* |
| *FAM100A* | *-0.59458* | *7.485596* | *-4.37706* | *3.72E-05* | *0.000619* | *1.974536* |
| *LOC643336* | *-0.59473* | *8.258846* | *-5.23489* | *1.38E-06* | *7.15E-05* | *5.088579* |
| *VSTM2L* | *-0.59693* | *8.382699* | *-6.26758* | *1.92E-08* | *5.20E-06* | *9.156487* |
| *VPS35* | *-0.59761* | *10.22394* | *-4.07289* | *0.000111* | *0.001323* | *0.947595* |
| *PTH2R* | *-0.59803* | *7.727303* | *-5.3842* | *7.55E-07* | *5.02E-05* | *5.658347* |
| *PRKCE* | *-0.59944* | *7.929932* | *-4.47475* | *2.59E-05* | *0.000484* | *2.313712* |
| *PLCXD3* | *-0.59996* | *8.044038* | *-3.64995* | *0.000474* | *0.003854* | *-0.39995* |
| *KIAA0513* | *-0.60065* | *10.06479* | *-4.92518* | *4.67E-06* | *0.000154* | *3.931004* |
| *CYP4X1* | *-0.60155* | *8.627331* | *-5.01769* | *3.25E-06* | *0.00012* | *4.273139* |
| *NPTN* | *-0.60242* | *10.89347* | *-4.45776* | *2.76E-05* | *0.000503* | *2.254412* |
| *CISD1* | *-0.60297* | *10.80776* | *-6.01661* | *5.55E-08* | *1.01E-05* | *8.143296* |
| *CDK5R1* | *-0.60321* | *10.5204* | *-4.36699* | *3.86E-05* | *0.000632* | *1.939817* |
| *SLC8A2* | *-0.60378* | *9.785929* | *-4.50173* | *2.34E-05* | *0.000452* | *2.408175* |
| *KCNK1* | *-0.60741* | *8.959933* | *-3.93772* | *0.000179* | *0.001866* | *0.506268* |
| *ATP6V1D* | *-0.60797* | *10.90138* | *-5.4865* | *4.99E-07* | *3.79E-05* | *6.052775* |
| *ATP1A1* | *-0.60859* | *10.47457* | *-4.53513* | *2.07E-05* | *0.000419* | *2.525519* |
| *SNCB* | *-0.61032* | *7.667275* | *-5.50803* | *4.57E-07* | *3.62E-05* | *6.136183* |
| *LRP11* | *-0.61057* | *8.92459* | *-3.74379* | *0.000347* | *0.003046* | *-0.10958* |
| *ERC2* | *-0.61142* | *8.615068* | *-3.61403* | *0.000534* | *0.004216* | *-0.50973* |
| *LPHN1* | *-0.61214* | *8.46604* | *-4.41275* | *3.26E-05* | *0.000569* | *2.097933* |
| *THY1* | *-0.61399* | *11.49455* | *-5.29217* | *1.09E-06* | *6.14E-05* | *5.306306* |
| *ADCY1* | *-0.61503* | *10.2952* | *-4.64851* | *1.35E-05* | *0.00031* | *2.927573* |
| *MORF4L2* | *-0.61554* | *9.34646* | *-3.58971* | *0.000579* | *0.004486* | *-0.58363* |
| *ATP2A2* | *-0.61563* | *10.29081* | *-5.45833* | *5.59E-07* | *4.12E-05* | *5.943854* |
| *ITPR1* | *-0.61647* | *9.901589* | *-3.34474* | *0.001272* | *0.008119* | *-1.30791* |
| *AP3B2* | *-0.61727* | *8.746722* | *-4.00007* | *0.000144* | *0.001597* | *0.708665* |
| *ABHD7* | *-0.61772* | *8.182332* | *-4.57331* | *1.79E-05* | *0.00038* | *2.660285* |
| *SCG3* | *-0.6188* | *9.998744* | *-6.06524* | *4.52E-08* | *8.96E-06* | *8.338563* |
| *SLC17A6* | *-0.62085* | *8.526614* | *-3.87097* | *0.000225* | *0.002217* | *0.29196* |
| *OXR1* | *-0.62105* | *10.75355* | *-4.06048* | *0.000116* | *0.001362* | *0.906672* |
| *MRPS30* | *-0.62119* | *8.112047* | *-5.84689* | *1.13E-07* | *1.61E-05* | *7.466141* |
| *SULT4A1* | *-0.62129* | *7.927112* | *-4.39382* | *3.50E-05* | *0.000594* | *2.032407* |
| *FLJ10781* | *-0.62369* | *9.720379* | *-3.84521* | *0.000246* | *0.002366* | *0.209902* |
| *NCOA7* | *-0.62483* | *10.15399* | *-3.58408* | *0.000589* | *0.004555* | *-0.60068* |
| *C2ORF55* | *-0.62563* | *10.25927* | *-4.23623* | *6.21E-05* | *0.000887* | *1.493418* |
| *LOC652900* | *-0.6262* | *7.285138* | *-5.27961* | *1.15E-06* | *6.35E-05* | *5.258466* |
| *SCOC* | *-0.62691* | *10.07445* | *-5.29693* | *1.07E-06* | *6.06E-05* | *5.324446* |
| *TMEM14A* | *-0.62787* | *8.71374* | *-4.09304* | *0.000104* | *0.001259* | *1.014187* |
| *ATP6V1E1* | *-0.63143* | *11.75002* | *-6.40158* | *1.08E-08* | *3.54E-06* | *9.702639* |
| *GABRA5* | *-0.6331* | *7.70346* | *-3.90724* | *0.000198* | *0.002014* | *0.408113* |
| *MICAL2* | *-0.63356* | *8.032337* | *-4.31522* | *4.66E-05* | *0.000718* | *1.762097* |
| *STAMBPL1* | *-0.63369* | *9.012228* | *-4.42378* | *3.13E-05* | *0.000551* | *2.136214* |
| *PGRMC1* | *-0.63562* | *10.43447* | *-4.20271* | *7.00E-05* | *0.000958* | *1.380323* |
| *AASDHPPT* | *-0.63613* | *9.160933* | *-3.85297* | *0.000239* | *0.002314* | *0.234594* |
| *GOLSYN* | *-0.63697* | *10.56376* | *-4.33026* | *4.41E-05* | *0.000692* | *1.8136* |
| *RNF175* | *-0.63841* | *8.51936* | *-4.84264* | *6.43E-06* | *0.000188* | *3.628451* |
| *FAM19A2* | *-0.6393* | *7.577754* | *-4.03535* | *0.000127* | *0.001452* | *0.824052* |
| *APLP1* | *-0.64019* | *9.350087* | *-3.89497* | *0.000207* | *0.002079* | *0.368743* |
| *PPM2C* | *-0.6413* | *8.080848* | *-4.05521* | *0.000118* | *0.001381* | *0.88931* |
| *PEX11B* | *-0.6413* | *9.562644* | *-6.03377* | *5.16E-08* | *9.80E-06* | *8.212154* |
| *AMPH* | *-0.64254* | *8.707013* | *-3.80669* | *0.00028* | *0.002598* | *0.08788* |
| *LINGO1* | *-0.64504* | *9.263406* | *-6.30262* | *1.65E-08* | *4.64E-06* | *9.29896* |
| *AK5* | *-0.64557* | *9.51006* | *-3.51424* | *0.00074* | *0.005383* | *-0.81069* |
| *LDOC1* | *-0.64767* | *8.628663* | *-4.24752* | *5.96E-05* | *0.000861* | *1.531644* |
| *YWHAE* | *-0.64768* | *8.039153* | *-4.21312* | *6.75E-05* | *0.000932* | *1.415392* |
| *C2ORF30* | *-0.64837* | *9.489794* | *-5.70866* | *2.00E-07* | *2.16E-05* | *6.919869* |
| *SATB2* | *-0.64914* | *9.428241* | *-3.45943* | *0.000883* | *0.00613* | *-0.97343* |
| *DDX24* | *-0.64963* | *9.501515* | *-4.69469* | *1.13E-05* | *0.000276* | *3.092894* |
| *LOC440928* | *-0.65004* | *8.817731* | *-4.32288* | *4.53E-05* | *0.000705* | *1.788322* |
| *VDAC1* | *-0.65144* | *10.92921* | *-4.5959* | *1.65E-05* | *0.000356* | *2.740335* |
| *C6ORF117* | *-0.6515* | *8.093662* | *-3.76292* | *0.000325* | *0.002909* | *-0.04976* |
| *WDR47* | *-0.6518* | *8.24333* | *-4.24164* | *6.09E-05* | *0.000874* | *1.51173* |
| *RTN4* | *-0.65532* | *11.25194* | *-5.44032* | *6.01E-07* | *4.33E-05* | *5.874359* |
| *TMEM132D* | *-0.6559* | *8.983158* | *-5.14458* | *1.97E-06* | *8.91E-05* | *4.747533* |
| *KRT222* | *-0.65849* | *8.155813* | *-5.64859* | *2.57E-07* | *2.51E-05* | *6.684042* |
| *ATL1* | *-0.65861* | *9.07141* | *-4.5552* | *1.92E-05* | *0.000397* | *2.596298* |
| *KIFAP3* | *-0.65907* | *10.465* | *-5.60088* | *3.12E-07* | *2.84E-05* | *6.497477* |
| *CBLN2* | *-0.66005* | *8.135804* | *-4.74521* | *9.35E-06* | *0.000243* | *3.274775* |
| *PKM2* | *-0.66031* | *8.733107* | *-4.09794* | *0.000102* | *0.001244* | *1.030419* |
| *PCLO* | *-0.66093* | *8.11846* | *-3.85341* | *0.000239* | *0.002312* | *0.235989* |
| *HS.7093* | *-0.66586* | *8.27296* | *-3.77152* | *0.000316* | *0.002844* | *-0.02279* |
| *CCKBR* | *-0.66687* | *7.88413* | *-6.61266* | *4.37E-09* | *2.07E-06* | *10.56944* |
| *SNAP25* | *-0.66721* | *13.31441* | *-3.94349* | *0.000175* | *0.001837* | *0.524935* |
| *RALYL* | *-0.67262* | *10.28558* | *-3.72962* | *0.000363* | *0.00316* | *-0.15376* |
| *ATP6AP2* | *-0.67298* | *11.12624* | *-4.87026* | *5.78E-06* | *0.000176* | *3.729397* |
| *XK* | *-0.67322* | *7.607895* | *-4.81582* | *7.13E-06* | *0.000201* | *3.530719* |
| *DRD1IP* | *-0.67383* | *11.04718* | *-5.62913* | *2.78E-07* | *2.65E-05* | *6.607858* |
| *HS.294103* | *-0.67434* | *9.797738* | *-5.10409* | *2.31E-06* | *9.75E-05* | *4.59554* |
| *KIAA1467* | *-0.67537* | *8.045323* | *-5.40805* | *6.85E-07* | *4.78E-05* | *5.750041* |
| *TBC1D9* | *-0.67562* | *8.909964* | *-4.78685* | *7.97E-06* | *0.000217* | *3.425467* |
| *RICS* | *-0.6768* | *10.47907* | *-4.77115* | *8.46E-06* | *0.000227* | *3.368582* |
| *NDFIP1* | *-0.67742* | *9.716358* | *-5.64337* | *2.62E-07* | *2.55E-05* | *6.66363* |
| *PRKCG* | *-0.67772* | *7.303894* | *-6.69051* | *3.12E-09* | *1.83E-06* | *10.89095* |
| *LOC642489* | *-0.67891* | *9.845096* | *-5.06256* | *2.73E-06* | *0.000108* | *4.440263* |
| *MAP2K1* | *-0.67909* | *9.992206* | *-3.90669* | *0.000199* | *0.002017* | *0.406341* |
| *MAGEE1* | *-0.67939* | *9.553193* | *-4.83289* | *6.67E-06* | *0.000192* | *3.592888* |
| *NMNAT2* | *-0.68195* | *8.254786* | *-5.22501* | *1.43E-06* | *7.25E-05* | *5.051144* |
| *ZNF25* | *-0.68251* | *9.793682* | *-5.51567* | *4.43E-07* | *3.52E-05* | *6.165821* |
| *FGF12* | *-0.68554* | *8.393974* | *-5.45097* | *5.76E-07* | *4.21E-05* | *5.915422* |
| *TMEM178* | *-0.68802* | *10.08437* | *-4.5502* | *1.96E-05* | *0.000401* | *2.578638* |
| *MOAP1* | *-0.68964* | *10.42258* | *-4.55518* | *1.92E-05* | *0.000397* | *2.596219* |
| *BASP1* | *-0.69129* | *12.54994* | *-4.64362* | *1.38E-05* | *0.000313* | *2.910139* |
| *BRWD1* | *-0.69389* | *8.854305* | *-6.48669* | *7.52E-09* | *2.92E-06* | *10.05125* |
| *OLFM3* | *-0.69576* | *7.573652* | *-4.97148* | *3.90E-06* | *0.000137* | *4.101857* |
| *SLITRK1* | *-0.69656* | *8.314763* | *-5.18308* | *1.69E-06* | *8.18E-05* | *4.892593* |
| *ELOVL4* | *-0.7012* | *9.71987* | *-4.79326* | *7.77E-06* | *0.000215* | *3.448707* |
| *CCK* | *-0.70138* | *9.779678* | *-4.37198* | *3.79E-05* | *0.000626* | *1.957002* |
| *CAMK1G* | *-0.70472* | *7.887741* | *-6.22995* | *2.25E-08* | *5.90E-06* | *9.003757* |
| *SH3GL2* | *-0.70662* | *11.72723* | *-4.19378* | *7.23E-05* | *0.00098* | *1.350268* |
| *TUBB4* | *-0.70689* | *10.40617* | *-3.64554* | *0.000481* | *0.003896* | *-0.41346* |
| *VIP* | *-0.70721* | *8.244895* | *-6.06617* | *4.50E-08* | *8.96E-06* | *8.34232* |
| *CRYM* | *-0.70761* | *7.543043* | *-5.95373* | *7.23E-08* | *1.23E-05* | *7.89163* |
| *TOMM20* | *-0.70847* | *11.16565* | *-5.42722* | *6.34E-07* | *4.55E-05* | *5.823834* |
| *SLC39A10* | *-0.70977* | *8.931107* | *-4.55574* | *1.92E-05* | *0.000396* | *2.598207* |
| *TCEAL6* | *-0.70997* | *8.631634* | *-5.36054* | *8.31E-07* | *5.39E-05* | *5.567609* |
| *YWHAZ* | *-0.71134* | *10.58242* | *-5.17216* | *1.77E-06* | *8.34E-05* | *4.851407* |
| *LOC387856* | *-0.71515* | *8.031168* | *-6.67101* | *3.40E-09* | *1.96E-06* | *10.81033* |
| *G3BP2* | *-0.71716* | *8.605948* | *-3.89096* | *0.00021* | *0.002103* | *0.355893* |
| *TSPAN7* | *-0.71788* | *12.59064* | *-5.83643* | *1.18E-07* | *1.66E-05* | *7.424624* |
| *FLJ33996* | *-0.72039* | *9.007955* | *-4.66423* | *1.27E-05* | *0.000298* | *2.983746* |
| *FBXL2* | *-0.72235* | *9.123552* | *-4.89342* | *5.28E-06* | *0.000165* | *3.814258* |
| *CHN1* | *-0.72333* | *11.63666* | *-5.50347* | *4.65E-07* | *3.66E-05* | *6.118521* |
| *SLC6A17* | *-0.72369* | *8.417522* | *-5.90351* | *8.92E-08* | *1.38E-05* | *7.691289* |
| *SCN3B* | *-0.72441* | *8.12023* | *-4.91207* | *4.91E-06* | *0.000159* | *3.88278* |
| *CDH13* | *-0.72676* | *7.990631* | *-4.71551* | *1.05E-05* | *0.000262* | *3.167742* |
| *CIRBP* | *-0.72712* | *10.56797* | *-5.39075* | *7.35E-07* | *4.92E-05* | *5.683535* |
| *ELAVL4* | *-0.72746* | *8.404476* | *-4.2975* | *4.97E-05* | *0.000753* | *1.701559* |
| *D4S234E* | *-0.73016* | *9.979172* | *-4.78705* | *7.96E-06* | *0.000217* | *3.426206* |
| *TMEM130* | *-0.73171* | *9.100806* | *-5.2834* | *1.13E-06* | *6.29E-05* | *5.272919* |
| *SNCA* | *-0.73389* | *8.646823* | *-4.01288* | *0.000137* | *0.001546* | *0.750495* |
| *REEP1* | *-0.73446* | *9.629559* | *-4.74947* | *9.19E-06* | *0.00024* | *3.29016* |
| *NECAP1* | *-0.74005* | *9.925673* | *-4.93284* | *4.53E-06* | *0.000151* | *3.959202* |
| *TUBB3* | *-0.74019* | *9.581959* | *-5.40895* | *6.83E-07* | *4.78E-05* | *5.7535* |
| *HS.155736* | *-0.74364* | *10.21803* | *-3.71055* | *0.000387* | *0.003321* | *-0.21301* |
| *ITFG1* | *-0.74526* | *8.386297* | *-4.45641* | *2.77E-05* | *0.000505* | *2.249701* |
| *PRKCB1* | *-0.74595* | *9.801557* | *-4.69452* | *1.13E-05* | *0.000276* | *3.092284* |
| *NAP1L2* | *-0.74715* | *7.827934* | *-4.55033* | *1.95E-05* | *0.000401* | *2.579102* |
| *ZNF365* | *-0.74754* | *8.951984* | *-4.70924* | *1.07E-05* | *0.000267* | *3.145166* |
| *ATP6V1B2* | *-0.75067* | *11.9082* | *-5.14285* | *1.98E-06* | *8.95E-05* | *4.74103* |
| *PCSK1N* | *-0.75106* | *8.657717* | *-4.14888* | *8.49E-05* | *0.001102* | *1.199852* |
| *C11ORF87* | *-0.75386* | *8.311263* | *-4.84335* | *6.41E-06* | *0.000188* | *3.631044* |
| *LRFN5* | *-0.75475* | *8.634314* | *-4.28933* | *5.12E-05* | *0.000771* | *1.673716* |
| *HMP19* | *-0.7553* | *9.697096* | *-5.63138* | *2.76E-07* | *2.64E-05* | *6.616695* |
| *CKMT1B* | *-0.75757* | *8.738712* | *-3.99969* | *0.000144* | *0.001599* | *0.70742* |
| *CPLX1* | *-0.75811* | *10.41741* | *-4.90554* | *5.04E-06* | *0.000161* | *3.858753* |
| *GABARAPL1* | *-0.7582* | *10.57755* | *-5.31747* | *9.88E-07* | *5.75E-05* | *5.402821* |
| *CPNE4* | *-0.75863* | *8.444078* | *-3.9469* | *0.000173* | *0.001822* | *0.535943* |
| *HS.538259* | *-0.75868* | *9.309514* | *-5.22647* | *1.42E-06* | *7.25E-05* | *5.05666* |
| *CYP26B1* | *-0.76066* | *8.789861* | *-4.45631* | *2.77E-05* | *0.000505* | *2.249378* |
| *CADPS* | *-0.76299* | *8.455934* | *-4.46392* | *2.70E-05* | *0.000497* | *2.275915* |
| *C12ORF53* | *-0.76686* | *8.185596* | *-4.41045* | *3.29E-05* | *0.000572* | *2.089986* |
| *B3GNT6* | *-0.76712* | *9.460354* | *-4.98828* | *3.65E-06* | *0.00013* | *4.164016* |
| *ENO2* | *-0.77207* | *10.87776* | *-5.09024* | *2.44E-06* | *0.000101* | *4.543672* |
| *MGC42367* | *-0.77317* | *10.63878* | *-4.9238* | *4.69E-06* | *0.000155* | *3.925923* |
| *DNM3* | *-0.77392* | *10.31682* | *-5.35204* | *8.60E-07* | *5.43E-05* | *5.535023* |
| *HS.576106* | *-0.77645* | *9.985081* | *-4.35687* | *4.00E-05* | *0.00065* | *1.904987* |
| *HSPB3* | *-0.7772* | *7.846604* | *-8.26389* | *3.04E-12* | *9.64E-08* | *17.51884* |
| *PPP3CB* | *-0.77909* | *10.60862* | *-5.11788* | *2.19E-06* | *9.42E-05* | *4.647254* |
| *UBE2N* | *-0.78193* | *10.31295* | *-5.94519* | *7.49E-08* | *1.23E-05* | *7.857504* |
| *CACNG3* | *-0.78457* | *9.848689* | *-5.70979* | *1.99E-07* | *2.16E-05* | *6.924312* |
| *HS.553187* | *-0.78614* | *8.358232* | *-4.11405* | *9.62E-05* | *0.001202* | *1.083863* |
| *NECAB1* | *-0.78748* | *8.430571* | *-3.83665* | *0.000253* | *0.002415* | *0.182725* |
| *RASL11B* | *-0.78829* | *8.88792* | *-4.36963* | *3.82E-05* | *0.000629* | *1.948908* |
| *PREPL* | *-0.78916* | *10.72202* | *-3.895* | *0.000207* | *0.002079* | *0.368845* |
| *SC5DL* | *-0.78954* | *9.074604* | *-5.35175* | *8.61E-07* | *5.43E-05* | *5.533908* |
| *NCALD* | *-0.79312* | *10.53134* | *-6.73035* | *2.63E-09* | *1.73E-06* | *11.05584* |
| *HOPX* | *-0.79509* | *9.590508* | *-6.17921* | *2.79E-08* | *6.65E-06* | *8.798242* |
| *CALY* | *-0.79628* | *7.74474* | *-5.6209* | *2.88E-07* | *2.67E-05* | *6.575673* |
| *GAD2* | *-0.7964* | *7.775346* | *-5.52524* | *4.26E-07* | *3.46E-05* | *6.202983* |
| *NSF* | *-0.79709* | *9.85244* | *-4.91012* | *4.95E-06* | *0.000159* | *3.87561* |
| *BEX4* | *-0.79945* | *10.403* | *-4.71081* | *1.07E-05* | *0.000266* | *3.150834* |
| *TMEM155* | *-0.80109* | *8.327165* | *-4.21781* | *6.63E-05* | *0.000922* | *1.431201* |
| *SLITRK4* | *-0.80169* | *9.178456* | *-4.66141* | *1.29E-05* | *0.0003* | *2.973654* |
| *HS.31961* | *-0.80439* | *9.838122* | *-4.15629* | *8.27E-05* | *0.00108* | *1.224607* |
| *HPCA* | *-0.80468* | *8.562856* | *-5.46765* | *5.38E-07* | *4.01E-05* | *5.979885* |
| *MKL2* | *-0.80505* | *9.623851* | *-4.95393* | *4.17E-06* | *0.000143* | *4.036984* |
| *PNMAL1* | *-0.81793* | *9.649908* | *-4.16841* | *7.92E-05* | *0.001047* | *1.265169* |
| *SCG5* | *-0.82521* | *11.83122* | *-4.93716* | *4.45E-06* | *0.00015* | *3.97512* |
| *COPG2IT1* | *-0.8254* | *9.686308* | *-4.37797* | *3.70E-05* | *0.000618* | *1.977671* |
| *PFN2* | *-0.82902* | *10.17677* | *-6.6921* | *3.10E-09* | *1.83E-06* | *10.89754* |
| *CKMT1A* | *-0.83272* | *9.240557* | *-4.02938* | *0.00013* | *0.001478* | *0.804487* |
| *RIMBP2* | *-0.83303* | *8.409354* | *-5.06686* | *2.68E-06* | *0.000107* | *4.456277* |
| *DACH2* | *-0.83656* | *7.969208* | *-4.84747* | *6.31E-06* | *0.000186* | *3.646097* |
| *C20ORF103* | *-0.83864* | *9.996985* | *-4.29157* | *5.08E-05* | *0.000765* | *1.681338* |
| *LPPR4* | *-0.84143* | *9.154987* | *-4.60369* | *1.60E-05* | *0.000349* | *2.767983* |
| *PPP3R1* | *-0.84254* | *9.954937* | *-4.64249* | *1.38E-05* | *0.000314* | *2.906106* |
| *BEX1* | *-0.84349* | *11.28584* | *-5.52311* | *4.29E-07* | *3.48E-05* | *6.194689* |
| *RCAN2* | *-0.84487* | *11.02079* | *-5.35332* | *8.55E-07* | *5.43E-05* | *5.539947* |
| *GNG2* | *-0.84504* | *8.319097* | *-3.88296* | *0.000216* | *0.00215* | *0.330282* |
| *SYN2* | *-0.84743* | *9.589748* | *-4.37157* | *3.79E-05* | *0.000626* | *1.955599* |
| *NUDT11* | *-0.84873* | *9.183327* | *-4.68857* | *1.16E-05* | *0.000281* | *3.070946* |
| *ADCYAP1* | *-0.85372* | *7.388649* | *-7.34608* | *1.78E-10* | *5.13E-07* | *13.62932* |
| *SNX10* | *-0.85576* | *8.550443* | *-4.57237* | *1.80E-05* | *0.000381* | *2.656976* |
| *EPB41L3* | *-0.85635* | *10.22865* | *-4.40229* | *3.39E-05* | *0.000583* | *2.061708* |
| *GOT1* | *-0.86426* | *11.09465* | *-5.16228* | *1.84E-06* | *8.54E-05* | *4.814151* |
| *MYT1L* | *-0.86554* | *9.321906* | *-4.06514* | *0.000114* | *0.00135* | *0.922022* |
| *TSPYL1* | *-0.86732* | *10.35976* | *-4.36808* | *3.84E-05* | *0.000631* | *1.94356* |
| *ATP1B1* | *-0.86753* | *11.1363* | *-4.0368* | *0.000126* | *0.001448* | *0.828814* |
| *KIAA0748* | *-0.8715* | *8.30043* | *-4.70052* | *1.11E-05* | *0.000273* | *3.113847* |
| *ANO3* | *-0.87275* | *9.063037* | *-4.37978* | *3.68E-05* | *0.000616* | *1.983914* |
| *PRKCB* | *-0.87778* | *9.85377* | *-4.37071* | *3.80E-05* | *0.000627* | *1.952647* |
| *BEX2* | *-0.88237* | *10.92274* | *-5.50098* | *4.70E-07* | *3.68E-05* | *6.108889* |
| *PTPRN* | *-0.88451* | *7.891853* | *-6.03062* | *5.23E-08* | *9.82E-06* | *8.199515* |
| *GPRASP2* | *-0.88582* | *8.95337* | *-6.0341* | *5.16E-08* | *9.80E-06* | *8.213463* |
| *GAD1* | *-0.88762* | *8.787411* | *-5.4465* | *5.87E-07* | *4.25E-05* | *5.898201* |
| *NAPB* | *-0.89344* | *11.76816* | *-4.30871* | *4.77E-05* | *0.000731* | *1.739863* |
| *NRGN* | *-0.89922* | *11.14964* | *-5.22511* | *1.43E-06* | *7.25E-05* | *5.051537* |
| *EPHA4* | *-0.9003* | *9.065163* | *-4.33683* | *4.31E-05* | *0.000684* | *1.836136* |
| *LDB2* | *-0.90117* | *11.01612* | *-4.77556* | *8.32E-06* | *0.000224* | *3.384556* |
| *C2ORF80* | *-0.91382* | *9.552418* | *-5.77236* | *1.54E-07* | *1.86E-05* | *7.171* |
| *TAGLN3* | *-0.91395* | *8.405132* | *-4.69365* | *1.14E-05* | *0.000277* | *3.089158* |
| *SLC12A5* | *-0.92428* | *11.083* | *-4.06594* | *0.000114* | *0.001348* | *0.924655* |
| *ATP6V1G2* | *-0.92758* | *10.35006* | *-5.69254* | *2.14E-07* | *2.26E-05* | *6.856491* |
| *SYT13* | *-0.92826* | *10.10003* | *-4.47124* | *2.63E-05* | *0.000488* | *2.301478* |
| *HS.390250* | *-0.92885* | *8.838181* | *-5.39068* | *7.35E-07* | *4.92E-05* | *5.683265* |
| *PNMA2* | *-0.92922* | *11.40033* | *-4.75611* | *8.96E-06* | *0.000237* | *3.314163* |
| *SST* | *-0.931* | *7.436277* | *-5.62996* | *2.77E-07* | *2.65E-05* | *6.611118* |
| *ELMOD1* | *-0.93326* | *8.92108* | *-4.68488* | *1.18E-05* | *0.000283* | *3.057721* |
| *SLC30A3* | *-0.93391* | *9.737455* | *-6.65978* | *3.56E-09* | *1.98E-06* | *10.76391* |
| *SCN2B* | *-0.94185* | *9.20284* | *-5.39618* | *7.19E-07* | *4.87E-05* | *5.704378* |
| *SYT4* | *-0.94961* | *11.4824* | *-4.03562* | *0.000127* | *0.001452* | *0.824962* |
| *GABRG2* | *-0.95768* | *9.516728* | *-4.42518* | *3.11E-05* | *0.000549* | *2.141055* |
| *ZCCHC12* | *-0.95828* | *8.701959* | *-4.89983* | *5.15E-06* | *0.000163* | *3.837809* |
| *NELL1* | *-0.96409* | *8.50368* | *-4.56537* | *1.85E-05* | *0.000387* | *2.632232* |
| *BEX5* | *-0.97023* | *8.370697* | *-5.95719* | *7.12E-08* | *1.22E-05* | *7.905435* |
| *YWHAG* | *-0.97118* | *9.880792* | *-4.74894* | *9.21E-06* | *0.000241* | *3.288241* |
| *CBLN4* | *-0.97482* | *7.835127* | *-5.76598* | *1.58E-07* | *1.88E-05* | *7.145774* |
| *GLRB* | *-0.975* | *8.777143* | *-4.56718* | *1.83E-05* | *0.000385* | *2.638606* |
| *DCLK1* | *-0.98283* | *8.999497* | *-4.8969* | *5.21E-06* | *0.000164* | *3.827057* |
| *SV2B* | *-0.98388* | *10.02313* | *-4.3302* | *4.41E-05* | *0.000692* | *1.813383* |
| *STX1A* | *-0.98647* | *9.163908* | *-5.13417* | *2.05E-06* | *9.09E-05* | *4.70839* |
| *TUBB2A* | *-0.98822* | *10.93601* | *-5.83016* | *1.21E-07* | *1.67E-05* | *7.399767* |
| *SVOP* | *-0.99184* | *8.679816* | *-6.31486* | *1.57E-08* | *4.48E-06* | *9.348811* |
| *CREG2* | *-0.99253* | *8.568988* | *-5.19177* | *1.63E-06* | *8.01E-05* | *4.925389* |
| *DIRAS2* | *-0.99685* | *9.292738* | *-4.02591* | *0.000131* | *0.001493* | *0.793107* |
| *STXBP1* | *-1.00073* | *8.452554* | *-5.4026* | *7.01E-07* | *4.79E-05* | *5.729078* |
| *NAP1L5* | *-1.00932* | *9.268861* | *-6.50725* | *6.88E-09* | *2.80E-06* | *10.13562* |
| *LOC100128403* | *-1.01131* | *9.512671* | *-4.94912* | *4.25E-06* | *0.000145* | *4.019217* |
| *ST6GALNAC5* | *-1.0114* | *8.077296* | *-5.753* | *1.67E-07* | *1.94E-05* | *7.094558* |
| *TMEM16C* | *-1.01249* | *8.690009* | *-4.88798* | *5.39E-06* | *0.000167* | *3.794308* |
| *PAK1* | *-1.02362* | *9.186484* | *-5.22923* | *1.41E-06* | *7.21E-05* | *5.067144* |
| *SCG2* | *-1.02494* | *9.986859* | *-4.98587* | *3.68E-06* | *0.00013* | *4.155104* |
| *VSNL1* | *-1.02575* | *11.29571* | *-4.2342* | *6.25E-05* | *0.00089* | *1.486543* |
| *NAP1L3* | *-1.03428* | *10.40492* | *-4.41323* | *3.25E-05* | *0.000569* | *2.099609* |
| *SYP* | *-1.04983* | *9.677255* | *-5.01108* | *3.34E-06* | *0.000122* | *4.248589* |
| *INA* | *-1.05566* | *10.2811* | *-4.71081* | *1.07E-05* | *0.000266* | *3.150802* |
| *GABBR2* | *-1.05728* | *10.92308* | *-4.38511* | *3.61E-05* | *0.000608* | *2.002329* |
| *KIAA1107* | *-1.05985* | *9.062362* | *-5.24435* | *1.32E-06* | *6.95E-05* | *5.124479* |
| *C1ORF173* | *-1.06092* | *8.589625* | *-4.9335* | *4.52E-06* | *0.000151* | *3.961628* |
| *UCHL1* | *-1.06224* | *10.66556* | *-4.34931* | *4.12E-05* | *0.000661* | *1.878992* |
| *SERPINI1* | *-1.06652* | *9.930737* | *-4.5533* | *1.93E-05* | *0.000398* | *2.589576* |
| *RTN1* | *-1.07174* | *10.4994* | *-4.67754* | *1.21E-05* | *0.000288* | *3.031408* |
| *TSPAN13* | *-1.07652* | *9.480274* | *-4.39883* | *3.43E-05* | *0.000587* | *2.049737* |
| *HPRT1* | *-1.08149* | *9.607595* | *-5.04223* | *2.95E-06* | *0.000113* | *4.364457* |
| *PARM1* | *-1.08845* | *9.638128* | *-4.89285* | *5.29E-06* | *0.000165* | *3.812182* |
| *MAL2* | *-1.10477* | *8.076676* | *-5.22563* | *1.43E-06* | *7.25E-05* | *5.053493* |
| *STAT4* | *-1.10647* | *8.078502* | *-6.07307* | *4.38E-08* | *8.78E-06* | *8.370081* |
| *VAMP2* | *-1.10716* | *9.899353* | *-5.72511* | *1.87E-07* | *2.07E-05* | *6.984627* |
| *RGS7* | *-1.12169* | *9.185568* | *-5.67303* | *2.32E-07* | *2.40E-05* | *6.779895* |
| *NELL2* | *-1.1237* | *10.1732* | *-3.92358* | *0.000188* | *0.001938* | *0.460674* |
| *NEFM* | *-1.12543* | *10.09999* | *-4.2387* | *6.15E-05* | *0.000882* | *1.501778* |
| *EFCBP1* | *-1.14115* | *10.15427* | *-4.14836* | *8.51E-05* | *0.001103* | *1.198122* |
| *CAP2* | *-1.15354* | *10.96884* | *-4.83539* | *6.61E-06* | *0.000191* | *3.601984* |
| *DYNC1I1* | *-1.15429* | *9.542837* | *-5.08297* | *2.51E-06* | *0.000103* | *4.516496* |
| *STMN2* | *-1.16633* | *11.24692* | *-5.15997* | *1.85E-06* | *8.58E-05* | *4.805465* |
| *GABRA1* | *-1.17129* | *8.713184* | *-5.06205* | *2.73E-06* | *0.000108* | *4.438322* |
| *PCSK1* | *-1.2085* | *8.03285* | *-6.21077* | *2.44E-08* | *6.24E-06* | *8.925993* |
| *ENC1* | *-1.21331* | *11.14187* | *-4.25983* | *5.70E-05* | *0.000833* | *1.573386* |
| *NPTX2* | *-1.22864* | *9.417981* | *-5.86655* | *1.04E-07* | *1.51E-05* | *7.544229* |
| *CHGB* | *-1.39666* | *9.0677* | *-5.18984* | *1.65E-06* | *8.03E-05* | *4.91813* |
| *SYT1* | *-1.48683* | *9.537753* | *-4.5678* | *1.83E-05* | *0.000385* | *2.640796* |
| *RGS4* | *-1.51422* | *10.41443* | *-5.35919* | *8.35E-07* | *5.39E-05* | *5.562413* |
| *VGF* | *-1.59578* | *9.851801* | *-7.6438* | *4.78E-11* | *3.03E-07* | *14.88639* |

**Supplementary Figure 1.** Quality Control Preprocess of included data.


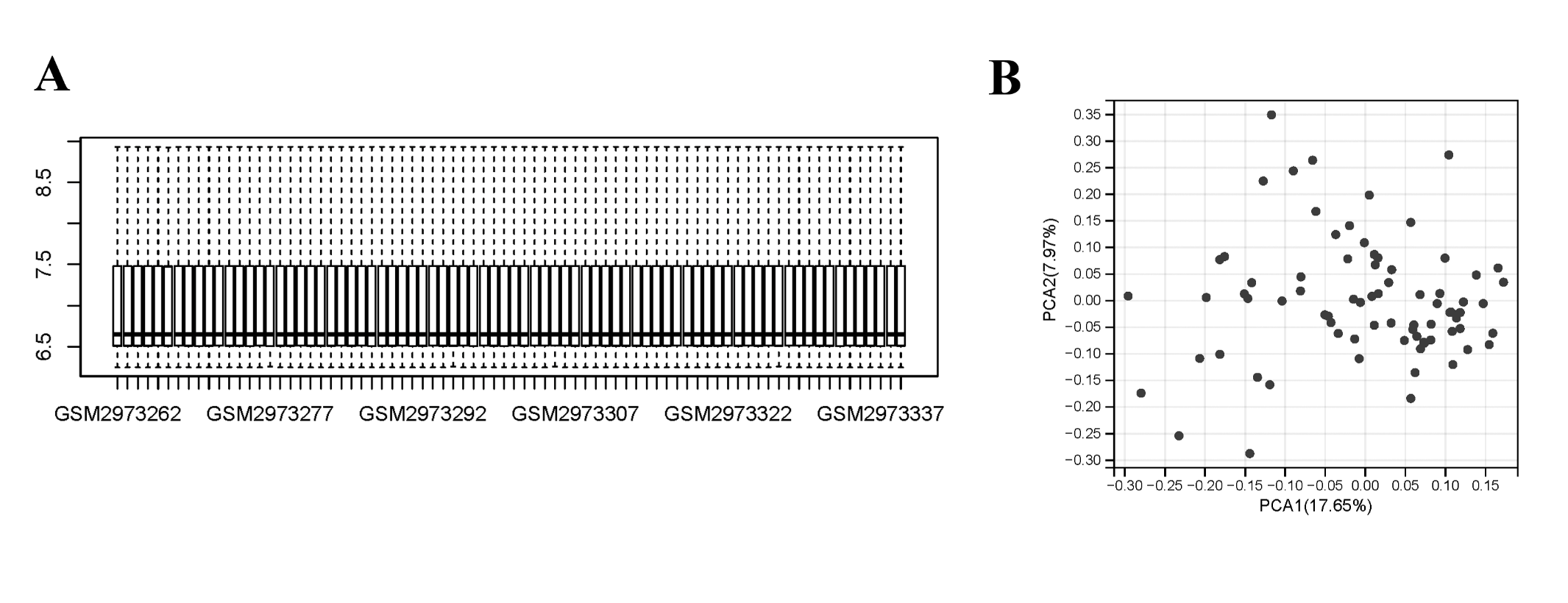

Supplement: Supplementary file 1 — Additional file 1: Supplementary Table 1. A total of 187 up-regulated genes and 280 down-regulated genes in Alzheimer’s disease. Supplementary Figure 1. Quality Control Preprocess of included data. [file 12883_2023_3503_MOESM1_ESM.docx]
